# Global disparities in patients with multiple myeloma: a rapid evidence assessment

**DOI:** 10.1038/s41408-023-00877-9

**Published:** 2023-07-18

**Authors:** Maria-Victoria Mateos, Sikander Ailawadhi, Luciano J. Costa, Shakira J. Grant, Lalit Kumar, Mohamad Mohty, Didem Aydin, Saad Z. Usmani

**Affiliations:** 1 University Hospital of Salamanca/IBSAL, Salamanca, Spain; 2 Division of Hematology/Oncology, Department of Medicine, Mayo Clinic, Jackson, FL USA; 3 O’Neal Comprehensive Cancer Center, University of Alabama at Birmingham, Birmingham, AL USA; 4 Department of Medicine, Division of Hematology, The University of North Carolina at Chapel Hill, Chapel Hill, NC USA; 5 Department of Medical Oncology, All India Institute of Medical Sciences, New Delhi, India; 6 Sorbonne University, Department of Hematology, Saint-Antoine Hospital, Paris, France; 7 Pfizer Pharmaceuticals, Istanbul, Turkey; 8 Myeloma Service, Department of Medicine, Memorial Sloan Kettering Cancer Center, New York, NY USA

**Keywords:** Myeloma, Myeloma, Quality of life

## Abstract

There are disparities in outcomes for patients with multiple myeloma (MM). We evaluated the influence of sociodemographic factors on global disparities in outcomes for patients with MM. This rapid evidence assessment (PROSPERO, CRD42021248461) followed PRISMA-P guidelines and used the PICOS framework. PubMed and Embase® were searched for articles in English from 2011 to 2021. The title, abstract, and full text of articles were screened according to inclusion/exclusion criteria. The sociodemographic factors assessed were age, sex, race/ethnicity, socioeconomic status, and geographic location. Outcomes were diagnosis, access to treatment, and patient outcomes. Of 84 articles included, 48 were US-based. Worldwide, increasing age and low socioeconomic status were associated with worse patient outcomes. In the US, men typically had worse outcomes than women, although women had poorer access to treatment, as did Black, Asian, and Hispanic patients. No consistent disparities due to sex were seen outside the US, and for most factors and outcomes, no consistent disparities could be identified globally. Too few studies examined disparities in diagnosis to draw firm conclusions. This first systematic analysis of health disparities in patients with MM identified specific populations affected, highlighting a need for additional research focused on assessing patterns, trends, and underlying drivers of disparities in MM.

## Introduction

Multiple myeloma (MM) is a hematological malignancy characterized by the clonal proliferation of malignant plasma cells in the bone marrow, the production of monoclonal protein, and multiple organ damage [1, 2]. MM is the second most common hematological malignancy [1, 2]. Globally, the incidence rate is ~2 per 100,000 people but varies considerably [3–5]. The highest rates are found in more developed nations such as the United States (US) and those of Australasia and Western Europe (≥4 cases per 100,000 people) [3, 4], probably due to greater awareness of the disease and better and more available diagnostic techniques [2]. Correspondingly, incidence rates are lower in less developed nations, such as those in Latin America, Asia, and Africa, where incidence rates are ≤2 cases per 100,000 people [3, 4, 6].

The etiology of MM is unknown and may be multifactorial [2]. Disparities exist in MM incidence and outcomes, including deaths. These disparities are partly caused by sociodemographic factors, such as age, sex, race/ethnicity, socioeconomic status, and geographic location, affecting healthcare utilization patterns, trends in treatment including access to clinical trials, and outcomes [7–10]. For instance, in the US, Black patients have a twofold increased risk of MM and are diagnosed with MM at younger ages compared with White patients [9–11] (note that descriptions of race/ethnicity throughout this article are based on those reported in the studies that met the inclusion criteria and were included for assessment). Moreover, despite recent therapeutic advances [12], improvements in outcomes have not been uniform among racial and ethnic minorities [13–16] or in patients diagnosed at an older age [14, 17]. Therefore, as improvements in the treatment of MM continue with the development of new agents or treatment paradigms, it is important to further identify disparities among patients and to inform and implement strategies to ensure equitable treatment for all, improve access to clinical trials, and improve standards of care [7, 10].

To the best of our knowledge, no systematic reviews have previously evaluated the global effects of sociodemographic factors on disparities in outcomes for patients with MM. Due to this lack of synthesized evidence, we conducted a rapid evidence assessment as the first systematic review of the topic in the published literature. The aim of the rapid evidence assessment was to highlight the impact of different sociodemographic factors on outcomes in patients with MM. Further, the identified disparities will inform areas for future research to improve access to equitable treatment, standards of care, and clinical trials.

## Materials and methods

The rapid evidence assessment was prospectively registered with the Prospective Register of Systematic Reviews (PROSPERO, registration number CRD42021248461; www.crd.york.ac.uk/prospero/) to avoid duplication and reduce potential reporting bias. The protocol followed the Preferred Reporting Items for Systematic Reviews and Meta-Analysis Protocol (PRISMA-P) guidelines [18] and defined all the processes and methodologies used. The assessment was conducted using the PICOS (population, intervention, comparison, outcome, and study type) framework for study selection and inclusion. Observational and real-world evidence studies were included in the meta-analysis. Preclinical studies, clinical studies, case studies, notes, commentaries, editorials, opinions, economic model studies, meta-analyses, reviews, and congress abstracts were excluded. Full inclusion and exclusion criteria for the studies included in this rapid evidence assessment are listed in Table 1.

**Table 1:** Study selection and inclusion criteria based on the PICOS framework.

	Inclusion criteria	Exclusion criteria
Population	Adults aged ≥18 years with MM	Studies in pediatric populations; studies where patients do not have MM; studies where outcomes for patients with MM are pooled with other conditions
Intervention/ comparator	Any	None
Outcomes	Diagnosis, access to treatment, and patient outcomes assessed by age, sex, race/ethnicity, socioeconomic status (income, education, insurance status, employment, housing), or geography (regional location within individual countries, rural, urban, or metropolitan location, distance from treatment center)	Outcomes of changeable factors such as comorbidities (i.e., anthropometric or behavioral factors such as obesity, alcohol, smoking, end-stage renal disease); performance status; occupational exposure; marital status; studies without mention of disparities or differences in outcomes by age, sex, race/ethnicity, socioeconomic status, or geography
Study types	Observational real-world evidence	Preclinical, clinical, and case studies; notes; commentaries; editorials; opinions; economic model studies; meta-analyses; reviews; congress abstracts
Other	Articles published in English from 2011 to 2021	Articles not in English or published before 2011; conference abstracts

*MM* multiple myeloma.

### Search strategy

Searches for published articles in English from 2011 to 2021 were conducted in PubMed and Embase®, excluding congress abstracts. A hand search of reference lists from relevant systematic literature reviews was also conducted to identify any articles that did not appear in the database searches. After duplicates were identified and removed, returned articles were screened for eligibility at level 1 (title and abstract) by a single reviewer according to the inclusion and exclusion criteria, and 20% of all screened articles were quality checked by a second reviewer. Discrepancies were discussed between reviewers until a consensus was reached, and if no consensus was reached the article was moved to level 2 screening. At level 2 screening, a single reviewer screened the full text of articles against the same inclusion and exclusion criteria, and 20% of all screened articles were quality checked by a second reviewer. Reasons for exclusion were recorded and cross-checked between the two reviewers, and a third reviewer was consulted for any discrepancies. The quality of the studies was assessed using the Newcastle–Ottawa Scale (NOS), with studies scored out of 9 for quality; high scores indicated a low risk of bias, and scores <5 indicated a poor-quality study with a high risk of bias.

### Data extraction and analysis

A single reviewer extracted data from studies that fulfilled all the inclusion criteria. All extracted data were then validated for accuracy by a second reviewer. The data elements extracted are listed in Supplementary Table 1. No inference analysis was conducted, and the data are descriptive only.

## Results

### Included studies, study characteristics, and quality assessment

**Fig. 1:**
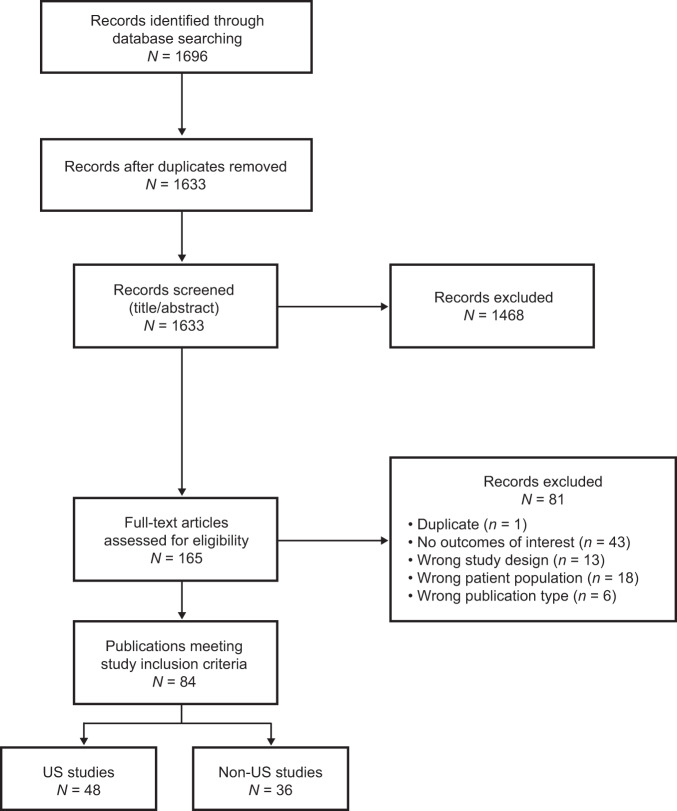
PRISMA flow diagram. *US* United States.

**Fig. 2:**
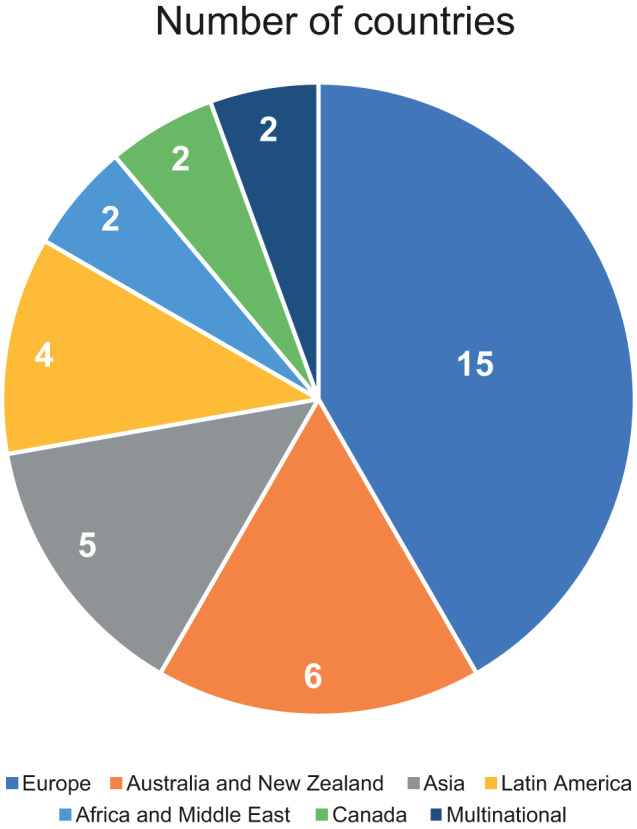
Geographic breakdown of non-US studies (*n* = 36). *US* United States.

**Fig. 3:**
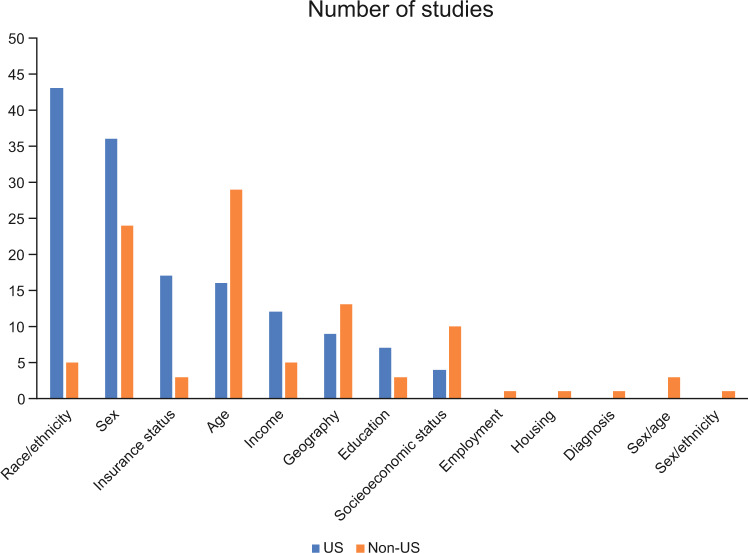
Factors assessed for disparity in MM outcomes. *MM* multiple myeloma, *US* United States.

The PRISMA flow diagram for the study is shown in Fig. 1. Overall, 1696 articles were identified, and after the removal of duplicates and exclusion by level 1 (title and abstract) and level 2 (full text) screening, 84 articles were identified as meeting the inclusion criteria. Of these 84 articles, 48 were studies in the US [14–16, 19–62], and 36 were studies outside the US (non-US) [63–97]. The full list of all 84 included studies is shown in Supplementary Table 2. Most of the 48 US-based studies used data from the Surveillance, Epidemiology, and End Results (SEER) database (*n* = 28), the National Cancer Database (NCDB, *n* = 8), and Medicare records (*n* = 8). The geographic breakdown of the 36 non-US studies is shown in Fig. 2. Most non-US studies were conducted in Europe (*n* = 15), followed by Australia and New Zealand (*n* = 6), Asia (*n* = 5), and Latin America (*n* = 4). The most common sociodemographic factors assessed for disparity are shown in Fig. 3. In the US studies, the most common factors assessed were race/ethnicity (*n* = 43), sex (*n* = 36), insurance status (*n* = 17), age (*n* = 16), and income (*n* = 12). In the non-US studies, the most common factors assessed were age (*n* = 29), sex (*n* = 24), geography (*n* = 13), and socioeconomic status (*n* = 10). Assessment of study quality using the NOS showed that all US studies and all but one non-US study had scores ≥5, indicating that no studies were poor quality or at high risk of bias. One non-US study [88] assessed using a modified NOS for cross-sectional studies had a score of 4. This study presented data from a survey of hematologists rather than from a patient registry, therefore the results carry a higher risk of bias. A higher proportion of US studies (94%) were considered high-quality and low-risk (NOS score of 8 or 9) compared with non-US studies (71%).

### Assessment of disparity

For the assessment of disparity, the following factors were selected: age, sex, race/ethnicity, socioeconomic status, and geography. These factors were assessed for the following outcomes: diagnosis, treatment access, and patient outcomes. Table 2 shows the number of studies evaluated for each factor by each outcome in the US studies and the studies from other countries/regions. Patient outcome was the most commonly assessed outcome (survival in 27 US and 21 non-US studies, and mortality in 13 US and 16 non-US studies), followed by access to treatment (24 US and 11 non-US studies). Few studies examined disparities in diagnosis (two US and four non-US studies). In the non-US studies, there was heterogeneity in the different measures of mortality, e.g., general mortality rate, early mortality, 5-year excess mortality rate ratio, and age-standardized mortality rate. Heterogeneity among both US and non-US studies was also noted regarding the different measures of access to treatment.

**Table 2:** Number of studies evaluating each factor assessed for disparity by outcome in the US and non-US studies.

Factors assessed for disparity	Outcome							
	Diagnosis		Access to treatment		Survival		Mortality	
	US	Non-US	US	Non-US	US	Non-US	US	Non-US
Total number of studies	2	4	24	11	27	21	13	16
Age	0	1	15	6	24	14	3	11
Sex	1	0	12	2	19	14	6	10
Race/ethnicity	2	0	19	2	24	4	11	1
Socioeconomic status	0	3	12	7	11	8	3	6
Geography	0	1	5	2	4	5	2	5

More than one factor may have been assessed per study.

*US* United States.

### Diagnosis

Only two US studies [14, 26] and four non-US studies (one each in France, Latin America, Mexico, and China) [84, 88, 93, 97] reported on disparities in diagnosis, therefore no clear patterns were observed. Increasing age affected the likelihood of receiving compliant care, including diagnosis [84], and female patients were older at diagnosis than males [14]. Hispanic patients and Black patients were significantly younger at diagnosis than White patients [14, 26]. Compared with patients receiving public healthcare, patients receiving private healthcare had a greater range and availability of diagnostic tests [88], and were less likely to be diagnosed with advanced-stage disease [93]. Patients with higher education levels had a shorter time to diagnosis than those with lower education levels [97]. Finally, patients who lived closer to the center where staging and prognostic procedures were performed were more likely to receive compliant care, including diagnosis [84].

### Access to treatment

Fifteen US studies and six non-US studies reported on age and disparities in access to treatment (Supplementary Table 3), with the majority showing that increasing age reduced access to treatment, including stem cell transplantation. Twelve US studies (Supplementary Table 4) and one study each in Europe and Canada reported on sex and access to treatment, most identifying better access to treatment in favor of men [31, 39, 40, 42, 43, 47, 54, 65, 80]. Access to stem cell transplantation was also reported to be worse in females [31, 40], except for one US study which reported that more Black women than Black men underwent autologous stem cell transplantation [26] and one Canadian study reporting no significant effect of sex on access to autologous stem cell transplantation [80]. For race/ethnicity and access to treatment, 19 US studies (Supplementary Table 5) and one study each in the United Kingdom and New Zealand were identified. Among the US studies, most reported worse access to treatment for Black [15, 21, 31, 32, 36, 37, 40, 42, 47, 54, 55], Asian [19, 31, 62], and Hispanic patients [15, 22, 31, 55] compared with White patients. In the US studies, there were trends suggesting that Hispanic patients and Black patients were less likely than White patients to receive stem cell transplantation [20, 22, 31, 32, 37, 40, 55] and that Black patients were more likely to experience delay in receiving stem cell transplantation compared with White patients [26]. In addition, one study in New Zealand observed significantly lower uptake of autologous stem cell transplantation in patients with Maori/Pasifika ethnicity compared with European or other ethnicities [66]. Twelve US studies and seven non-US studies reported on socioeconomic status and access to treatment (Supplementary Table 6), with mixed results. Lower socioeconomic status, whether defined by general socioeconomic status, household income, education level, or insurance status, was generally associated with worse access to treatment, including stem cell transplantation[27, 36, 37, 39, 40, 43, 50, 54, 58, 72, 80, 88, 93, 97], although this was not a universal finding [36, 42, 43, 47, 50, 54, 58, 60, 66, 96]. The effects of geography on access to treatment were reported in five US studies (Supplementary Table 7) and one study each in Canada and France [80, 84]. No consistent findings on differences in access to treatment, including stem cell transplantation, with respect to living in rural, urban, or metropolitan areas were found [40, 42, 43, 54, 80].

### Patient outcomes

Twenty-four US and 14 non-US studies reported on age and disparities in survival (Supplementary Table 8), and three US and 11 non-US studies on age and disparities in mortality (Supplementary Table 9). Most studies reported that survival and mortality worsened with increasing age [14, 16, 22, 23, 25, 27, 29, 30, 35, 37, 38, 40, 41, 43, 45, 46, 48, 51, 55–57, 59, 61, 66, 67, 69, 71, 73–75, 77, 80, 82, 83, 85–87, 89, 90, 96, 98].

Nineteen US and 14 non-US studies reported on sex and survival (Supplementary Table 10), and six US and 10 non-US studies on sex and mortality (Supplementary Table 11). Most studies reported no effect of sex on survival or mortality [16, 25, 27, 32, 34, 38, 45, 56, 59, 63, 66, 68, 69, 74, 75, 80, 84, 90, 92, 97]. However, a subset of studies reported more favorable survival in women [14, 22, 29, 40, 41, 43, 48, 55, 61], and mortality rates were generally higher in men [29, 44, 46, 67, 70, 77, 82, 89, 92, 94, 98, 99].

Twenty-four US and four non-US studies reported on race/ethnicity and survival (Supplementary Table 12), and 11 US studies (Supplementary Table 13) and one study in New Zealand on race/ethnicity and mortality. Overall, no clear patterns emerged. A large proportion of studies reported no effect of race/ethnicity on survival or mortality [20, 22–27, 30, 32, 34, 38, 41, 45, 46, 55–57, 61, 66, 72]. Several studies reported better survival (as measured by overall survival [OS], myeloma-specific survival, 1-year OS, 5-year OS, or relative survival) for Black/African American patients versus White patients [14, 20, 23, 24, 40, 48, 51, 90], although Black/African American patients may have higher mortality rates (as measured by excess mortality, rate ratio, mortality rate, or risk of dying) than White patients [16, 33, 37, 44, 49, 52]. Some studies reported worse survival or mortality for Hispanic versus White patients [14–16, 29, 53], but this was not universally reported [15, 20, 23, 30, 34, 43, 46, 48, 53]. Asian patients were generally reported to have better survival versus White, Black/African American, or Hispanic patients [14, 23, 45].

Eleven US and eight non-US studies reported on socioeconomic status and survival (Supplementary Table 14), and three US and six non-US studies on socioeconomic status and mortality (Supplementary Table 15). Lower socioeconomic status, whether defined generally or specifically in terms of income, education level, or insurance status, was associated with worse survival and mortality [23, 27, 29, 35, 37, 38, 40, 41, 43, 44, 48, 56, 63, 66, 69, 72, 78, 90, 91, 93, 97].

Four US and five non-US studies reported on geography and survival (Supplementary Table 16), and two US [44, 46] and five non-US studies (Supplementary Table 17) on geography and mortality. No clear patterns emerged. Differences in survival or mortality for patients in rural versus urban versus metropolitan areas were variable [40, 41, 43, 48, 63, 69, 80, 81, 97, 98]. Comparison of different regions within individual countries, such as the US, Canada, China, and New Zealand, demonstrated varying survival or mortality rates [46, 66, 77, 94].

## Discussion

In this rapid evidence assessment, we assessed disparities in MM on a global scale by examining variations in an array of sociodemographic factors such as age, sex, race/ethnicity, socioeconomic status, and geographic location on diagnosis, access to treatment, and patient outcomes. Published literature was assessed using a standardized, thorough, and transparent approach using the PICOS framework for study selection and inclusion. To the best of our knowledge, this is the first formal, comprehensive review of literature that has reported and compared disparities in MM at a global level. It highlights the heterogeneity of the data and the multifactorial nature of disparities in MM and identifies areas for future research to ensure that disparity among patients does not affect equitable treatment.

There were clear disparities in access to treatment and outcomes for some of the sociodemographic factors assessed, with agreement among studies indicative of a global problem. Increasing age was associated with worse access to treatment, and worse access typically occurred in Black, Asian, and Hispanic patients compared with White patients. In addition, Hispanic and Black patients in US studies were less likely to receive stem cell transplantation compared with White patients, and Black patients were more likely to experience delays in access to stem cell transplantation compared with White patients. Access to treatment was also generally worse for women. Increasing age was also associated with worse patient outcomes, as was lower socioeconomic status irrespective of how it was measured. The effect of age on patient outcomes is not necessarily surprising. MM usually affects older patients, and traditionally survival and mortality were worse for older patients because high-dose therapy followed by stem cell transplant was not a valid approach [100]. With the increasing availability of proteasome inhibitors, immunomodulatory drugs, and anti-CD38 therapies, the lack of barriers to their use in older patients, and the adoption of frailty-adapted therapy, the prognosis for older patients continues to improve [101]. Although survival for older patients still lags behind their younger counterparts [30, 102], the disparity in patient outcomes because of age may be potentially resolved in the future. Another clear finding was the disparity in access to treatment for women compared with men, further indicating the need for an increased focus on sex-stratified medicine [103].

Sometimes, no clear pattern could be observed among factors and outcomes. These inconsistent findings might be explained by heterogeneity among studies, for instance in outcome measures, study quality, database size, or type of analysis (e.g., univariate or multivariate), or the country(ies) involved in the study as reflected in patient populations or health systems. This inconsistency may also reflect the multifactorial nature of disparities in MM and the possibility that factors may be confounding and difficult to isolate. For instance, we should consider that identifying single predictive factors of disparity is difficult when it is likely that it is a combination of age, race/ethnicity, and low socioeconomic status, whether defined by income, education, or insurance status, that leads to lack of access to treatment or worse patient outcomes rather than each individual factor alone.

Some studies examining disparities due to race/ethnicity have previously demonstrated an effect of race/ethnicity [11, 13–16], but this has not been a universal finding [7, 10]. The results from our assessment further emphasize the variability among studies. As noted above, determining the contribution of a single factor such as race/ethnicity on any disparity is difficult because of the confounding nature of multiple, interacting factors. For instance, Black patients often face additional barriers in accessing MM care, leading to delayed diagnosis and later treatment initiation. Moreover, the biology of disease is an important consideration that may vary across different races and ethnicities. A recent study demonstrated superior survival in African American patients compared with White patients when both groups had equal access to healthcare [104], which may reflect differences in disease biology. Disease biology may also be an important consideration for other factors, such as age, and of particular relevance when considering treatment with immunomodulatory drugs.

This rapid evidence assessment is inherently limited by its descriptive nature. Searches were restricted to PubMed and Embase® between 2011 and 2021, and congress abstracts were not included. Only studies in the English language were included. Given the global nature of research into disparities in MM, relevant studies in other languages may have been missed. Data were descriptive only, and no inference analysis was conducted. Only a limited number of sociodemographic factors were assessed for disparity. Other anthropometric or behavioral factors, such as obesity, alcohol use, smoking, marital status, occupational exposure, disease stage, genetic factors, and comorbidities, were not examined and could be confounding factors for patient outcomes affecting the assessment of health disparities. Our study also found heterogeneity among studies in terms of measures for each outcome and type of analysis. Moreover, the US studies had to rely primarily on only a few databases (SEER, *n* = 28; NCDB, *n* = 8; Medicare records, *n* = 8) leading to possible patient overlap and duplication of populations.

This study identifies several areas for possible future research. Of the 84 studies that met the inclusion criteria, 63 (75.0%) were either in the US or Europe. Furthermore, for most outcomes, there was heterogeneity between studies, notably for measures of mortality and treatment access, that may benefit from standardization. New studies outside the US and Europe that utilize standardized outcomes and measures would not only enable the assessment of disparities on a global scale but also enable direct comparison among countries. We found clear disparities due to lower socioeconomic status, which is multifactorial in nature; and disparities due to variations in race/ethnicity may be indirectly affected by associated variations in socioeconomic status or access to treatment [8]. Some sociodemographic factors were assessed in this study, and confounding factors may also be pertinent for patient outcomes, such as those anthropometric or behavioral factors noted above [7]. This would help clinicians to further understand the complex multifactorial nature of MM where different factors may combine to affect patient outcomes, or different factors may influence each other. Given the well-documented improvements in survival over the past 10–20 years [105, 106], future research could focus on changes in disparity over time. Few studies in the US or elsewhere examined disparities in diagnosis, which is of particular importance given differences in the quality, availability, and delivery of diagnostic techniques among countries [2]. This study therefore identifies a clear need for increased research around disparities in diagnosis. Except for a few studies outside the US, for instance in New Zealand [66, 92], somewhat surprisingly the effects of race/ethnicity on disparities in MM have received little attention. Additional studies globally would improve our understanding of this phenomenon, and how to address it.

The disparities we have systematically identified in our rapid evidence assessment of global barriers to accessing treatment for patients with MM align with some of the previously mentioned barriers to accessing treatment and clinical trials such as age, sex, race/ethnicity, and socioeconomic status [7–10]. Improving access to clinical trials by overcoming some of these barriers would include designing more diverse clinical trials with evidence-based eligibility criteria that promote recruitment and retention, improving physician–patient communication, tailored patient education, and overcoming physical and transportation barriers to clinic visits through telemedicine and home visits [8]. Adopting some of these suggested changes to clinical trials could also improve access to MM treatment in general either directly, for example through improving physician–patient communication, or indirectly by increasing physician and patient confidence that new treatments are effective and appropriate for individual patients.

In conclusion, this study highlights specific populations of patients with MM that remain at a disadvantage and for whom there is potential scope for improvement in outcomes. The study also shows that data are heterogeneous and that certain factors uniformly cause disparity in access to treatment, namely being older, being female, or being Black, Asian, or Hispanic, whereas others are variable and multifactorial in nature, such as lower socioeconomic status. Acknowledging and addressing the causes and effects of disparities in patient outcomes may help to develop novel treatments or treatment strategies for MM, for instance through the enrollment of more diverse and representative patient populations in clinical trials [8], and to improve access to treatment and treatment facilities in the real world. Similarly, acknowledging and addressing the need for standardizing measures of mortality and treatment access across studies is important to improve the evaluation of access and outcomes across patient groups and treatment regimens. This rapid evidence assessment also highlights the need for more comprehensive evaluations of the barriers to treatment in MM. Ultimately, an improved understanding of disparities in MM should help to guide appropriate treatment choices, to ensure that there is equitable treatment for all and that patients derive maximum benefit.

## Supplementary information

Supplementary Tables 1–17

## Data Availability

Upon request, and subject to review, Pfizer will provide the data that support the findings of this study. Subject to certain criteria, conditions, and exceptions, Pfizer may also provide access to the related individual de-identified participant data. See https://www.pfizer.com/science/clinical-trials/trial-data-and-results for more information.
