# Supplementary material for: Global disparities in patients with multiple myeloma: a rapid evidence assessment
Source: Blood Cancer J. 2023 Jul 18;13(1):109. doi: 10.1038/s41408-023-00877-9 (PMC10352266; doi:10.1038/s41408-023-00877-9)
Supplement: Supplementary file 1 — Supplementary Tables 1–17 [file 41408_2023_877_MOESM1_ESM.pdf]

Global disparities in patients with multiple myeloma: a rapid evidence assessment

Maria-Victoria Mateos, Sikander Ailawadhi, Luciano J. Costa, Shakira J. Grant,

Lalit Kumar, Mohamad Mohty, Didem Aydin and Saad Z. Usmani

Supplementary Material – Table of contents

Item	Page
Supplementary Table 1: Data elements extracted from studies meeting inclusion criteria	2
Supplementary Table 2: List of studies meeting inclusion criteria	3
Supplementary Table 3: Age and disparities in access to treatment in US and non-US studies	8
Supplementary Table 4: Sex and disparities in access to treatment in US studies	10
Supplementary Table 5: Race/ethnicity and disparities in access to treatment in US studies	11
Supplementary Table 6: Socioeconomic status and disparities in access to treatment in US and non-US studies	13
Supplementary Table 7: Geography and disparities in access to treatment in US studies	15
Supplementary Table 8: Age and disparities in survival in US and non-US studies	16
Supplementary Table 9: Age and disparities in mortality in US and non-US studies	18
Supplementary Table 10: Sex and disparities in survival in US and non-US studies	19
Supplementary Table 11: Sex and disparities in mortality in US and non-US studies	21
Supplementary Table 12: Race/ethnicity and disparities in survival in US studies	22
Supplementary Table 13: Race/ethnicity and disparities in mortality in US studies	24
Supplementary Table 14: Socioeconomic status and disparities in survival in US and non-US studies	25
Supplementary Table 15: Socioeconomic status and disparities in mortality in US and non-US studies	27
Supplementary Table 16: Geography and disparities in survival in US and non-US studies	28
Supplementary Table 17: Geography and disparities in mortality in non-US studies	29
References	30

Supplementary Table 1. Data elements extracted from studies meeting inclusion criteria

Author, year, article type
Study sponsor, country, region
Study/data source name
Study design (longitudinal, cross-sectional)
Study period
Sample size
Disparities: age, sex, race/ethnicity, socioeconomic status, geography
Outcomes: diagnosis, access to treatment, patient outcomes
Summary of findings
Quality assessment of evidence and tool used

Supplementary Table 2. List of studies meeting inclusion criteria

Author, year	Country	Study population	Data source	Data period	Sample size analyzed (n)	Factors assessed
Afshar, 2020 [1]	Australia	Patient with 1st primary cancer (MM), age 15–99 y	Victorian Cancer Registry	2001–2015	5021	Age, sex, socioeconomic status, geography
Ailawadhi, 2012 [2]	US	Patients with primary MM	SEER Registry	1992–2007	37,963	Age, sex, race/ethnicity
Ailawadhi, 2016 [3]	US	Adults (18 y) diagnosed with MM	SEER Registry	1973–2011	NCI: 71,364<br>NCCN: 52,122	Race/ethnicity
Ailawadhi, 2017 [4]	US	All cases of primary MM	SEER Registry-Medicare	Diagnosed: 2007–2009<br>Follow-up to 2012	5338	Age, sex, race/ethnicity
Ailawadhi, 2018 [5]	US	All cases of primary MM reported, with full Medicare coverage	SEER Registry-Medicare	1991–2010	32,815	Age, sex, race/ethnicity, socioeconomic status
Ailawadhi, 2019 [6]	US	White, African American, and Hispanic patients with a confirmed MM diagnosis	SEER Registry-Medicare	2007–2013	4830	Race/ethnicity
Ailawadhi, 2019 [7]	US	Adult patients (>18–40 y) with confirmed MM diagnosis	SEER-18 Registry	1973–2014	1460	Sex, race/ethnicity
Ailawadhi, 2020 [8]	US	Adult patients aged ≥18 y who had symptomatic MM diagnosed within 2 mo before enrollment	Connect MM Registry	Cohort 1: 2009–2011;<br>Cohort 2: 2012–2016	2912	Age, sex, race/ethnicity
Ailawadhi, 2020 [9]	US	Adult patients aged ≥18 y who had symptomatic MM diagnosed within 2 mo before enrollment	Connect MM Registry	Cohort 1: 2009–2011;<br>Cohort 2: 2012–2016	2837	Race/ethnicity
Ailawadhi, 2021 [10]	US	Adults with a diagnosis of MM or osseous plasmacytoma or extraosseous plasmacytoma	NCDB Registry	2004–2013	101,919	Race/ethnicity
Auner, 2012 [11]	UK	Patients with 1st ASCT for MM	Medical records	1994–2009	363	Race/ethnicity
Auner, 2015 [12]	Europe	Patients with 1st AHCT for MM	EBMT	1991–2010	53,675	Age, sex
Bhatnagar, 2015 [13]	US	Patients with MM undergoing ASCT	Retrospective chart review, University of Maryland	2000–2013	453	Race/ethnicity
Chamoun, 2021 [14]	US	All patients diagnosed with MM between 2005 and 2014	NCDB Registry	2005–2014	115,099	Age, sex, race/ethnicity, socioeconomic status
Chan, 2020 [15]	New Zealand	Patients with MM	NZCR	2012–2016	1864	Age, sex, race/ethnicity, socioeconomic status, geography
Chang-Chan, 2021 [16]	Spain	Patients with MM	Grenada and Girona PBCRs; INE	2010–2016	654	Age, sex
Chen, 2016 [17]	Taiwan	Patients with NDMM	NHIRD	1997–2013	7285	Age, sex, socioeconomic status, geography

Chhabra, 2020 [18]	US	All MM patients aged ≥18 y who underwent HPC mobilization and collection for an upfront AHCT	Single-center chart review	2012–2017	400	Age
Costa, 2015 [19]	US	New cases of MM	SEER-18 Registry	2005–2009	22,462	Age, sex, race/ethnicity, socioeconomic status
Costa, 2016 [20]	US	Patients with MM diagnosed at age <65 y	SEER Registry	2007–2012	10,161	Age, sex, race/ethnicity, socioeconomic status
Costa, 2017 [21]	US	Patients with MM	SEER-13 Registry	1993–2012	34,505	Age, sex, race/ethnicity
Derman, 2020 [22]	US	Patients with newly diagnosed MM	MMRF CoMMpass Registry	2011–NR	639	Age, sex, race/ethnicity
DeSantis, 2016 [23]	US	All cancer patients in the US; subpopulation myeloma	SEER Registry	2008–2012	NR	Sex, race/ethnicity
Dhakal, 2020 [24]	US	Adults aged ≥18 y with MM who received TPE in the inpatient setting	NIS Registry	1993–2015	2008–2015: 561	Age, sex, race/ethnicity, socioeconomic status
El Husseiny, 2014 [25]	Egypt	Patients with MM	Medical records of participating institution	2000–2010	116	Age, sex
Evans, 2021 [26]	US	Patients with NDMM	NCDB Registry and a tertiary care center	NCDB cohort: 2004–2015; tertiary care center: 2005–2015	NCDB cohort: 122,858; tertiary care center: 2543	Age, socioeconomic status
Fakhri, 2018 [27]	US	Adults >65 y diagnosed with MM and enrolled in Medicare Parts A, B, and D	SEER Registry-Medicare	2007–2011	3814	Age, sex, race/ethnicity, socioeconomic status
Fiala, 2015 [28]	US	Adults aged >18 y diagnosed with MM	Washington University School of Medicine; SEER 18 Registry	2000–2009	WUSM: 562<br>SEER: 45,505	Age, race/ethnicity, socioeconomic status
Fiala, 2017 [29]	US	Patients with MM	SEER Registry-Medicare	2000–2011	20,916	Age, sex, race/ethnicity, socioeconomic status, geography
Fiala, 2020 [30]	US	Adults aged ≥70 y with NDMM	SEER Registry	2007–2013	70–79 yrs: 2933<br>80+ yrs: 2155	Age, sex, race/ethnicity, socioeconomic status
Fiala, 2020 [31]	US	Patients with myeloma	SEER Registry-Medicare	2007–2013	6272	Age, sex, race/ethnicity, socioeconomic status
Harwood, 2020 [32]	Australia	Patients with MM	Oncology Analysis System	1982–2014	6025	Age, sex, socioeconomic status, geography
Hsieh, 2019 [33]	US	Patients with active MM	NCDB Registry	Enrollment: 2004–2006<br>Follow-up to 2016	26,986	Age, sex, race/ethnicity, socioeconomic status, geography
Hsu, 2015 [34]	Taiwan	Patients with symptomatic MM	Medical records	2002–2015	460	Age, sex
Ilic, 2014 [35]	Serbia	Patients who died from MM	Statistical Office of the Republic of Serbia	1991–2010	2724	Age
Intzes, 2020 [36]	Greece	Patients with symptomatic MM	Medical records of participating institution	2005–2019	223	Race/ethnicity, socioeconomic status

Jayakrishnan, 2020 [37]	US	Patients treated within 120 d of MM diagnosis	NCDB registry	2004–2016	65,723 with MM Dx and treatment at reporting facility	Age, sex, race/ethnicity, socioeconomic status, geography
Jayakrishnan, 2021 [38]	US	Patients diagnosed with MM and treated with 1L systemic therapy	NCDB registry	2004–2016	Enrollment analysis group: 56,102<br>Survival analysis group: 50,543	Age, sex, race/ethnicity, socioeconomic status, geography
Jones, 2021 [39]	England	Patients aged 15–90 y diagnosed with MM	NCR and Analysis Service and NHS Hospital Episode Statistics database	1998–2015	2010–2014: 19,729	Age
Jurczyszyn, 2016 [40]	Multinational	Previously untreated patients aged 21–60 y with MM	Medical records of participating institutions	2000–2015	1089	Age, sex
Kamath, 2020 [41]	US	Patients with MM	SEER Registry; National Center for Health Statistics; The New York State Cancer Registry; 2010 US Census	1995–2016	53,599	Sex, race/ethnicity, socioeconomic status, geography
Kaya, 2012 [42]	US	MM diagnosis	SEER Registry	1997–2003 database; contains data for 1973–2003	40,294	Age, sex, race/ethnicity
Kim, 2014 [43]	Asia (China, Hong Kong, Japan, Korea, Singapore, Thailand)	Patients with symptomatic MM	Medical records/databases from participating tertiary centers	1986–2011	3405	Age, sex
Kumar, 2020 [44]	US	Patients with MM	NCDB Registry	2004–2015	74,722	Age, sex, race/ethnicity, socioeconomic status, geography
Kumar, 2021 [45]	US	Patients with newly diagnosed MM	SEER Registry	1975–2015	90,975	Age, sex, race/ethnicity, geography
Lin, 2019 [46]	Global	Patients with MM	GBD study 2017	2017	NR	Socioeconomic status
Liu, 2019 [47]	China	Patients with MM	China CDC	2006–2016	NR	Age, sex, geography
Mahumud, 2019 [48]	Australia	Patients with cancer	ABDS	2011–2015	NR	Socioeconomic status
Makhani, 2021 [49]	US	Adults aged >18 y with primary diagnosis of MM	SEER Registry	2007–2016	41,789	Age, sex, race/ethnicity, socioeconomic status, geography
Manyega, 2021 [50]	Kenya	Patients with MM	MTRH	2009–2019	221	Age, sex
Marron, 2018 [51]	US	Cohort of Black and White men and women from Pittsburgh, Pennsylvania and Memphis, Tennessee aged 70–79 y during recruitment in 1997 and 1998	Health ABC study cohort	Recruited 1997/1998<br>Follow-up: median 13.1 yrs	NR for MM cohort	Race/ethnicity
Mian, 2021 [52]	Canada	Patients with NDMM	Administrative healthcare data in Ontario, Canada	2007–2017	≤65: 3053<br>>65: 5788	Age, sex, socioeconomic status, geography

Ng, 2020 [53]	Australia	Patients with MM diagnosed in the Western Australia public hospital system	NR; hospital records from Western Australia public hospital system	2008–2018	568	Geography
Pan, 2021 [54]	US	Patients with MM who were treated with ASCT	Mount Sinai Hospital Registry	2011–2016	410	Age, sex, race/ethnicity, socioeconomic status
Pastor-Barriuso, 2014 [55]	Spain	Patients who died from MM	Spanish National Institute of Statistics	1952–2006	7801	Age, sex
Patel, 2020 [56]	US	US veterans aged ≥65 y	Veterans Administration Central Cancer Registry	1999–2014	3807	Age, race/ethnicity
Pinheiro, 2020 [57]	US	All cancer deaths from the 18 most common cancers	State's Department of Vital Statistics	2012–2017	NR for MM cohort	Sex, race/ethnicity
Pulte, 2012 [58]	US	All cancer patients in the US; subpopulation myeloma	SEER 9 Registry	1973–2007	1992–1996: 5584<br>2002–2006: 6351	Age, race/ethnicity
Pulte, 2014 [59]	US	Patients aged >15 y with first diagnosis of MM	SEER 13 Registry	1993–2009	26,391	Age, sex, race/ethnicity
Pulte, 2015 [60]	Germany	Patients aged 15–74 y with MM	12 German cancer registries	1997–2010	11,750	Age, sex
Puyade, 2018 [61]	France	Patients with MM, resident in region >6 mo, eligible for active treatment/ surveillance	Poitou-Charentes Cancer Registry	2008–2010	367	Age, sex, geography
Quaresma, 2015 [62]	UK	Patients with first, primary, invasive MM aged 15–99 y	NCR; WCISU	1971–2011	97,428	Age, sex
Radocha, 2019 [63]	Czech Republic	Patients >65 y with symptomatic NDMM	Czech RMG	2007–2016	1410	Age, sex
Riva, 2019 [64]	Uruguay	Patients with symptomatic MM	Medical records of participating institutions	2012–2015	222	Age
Riva, 2020 [65]	Latin America	Patients with MM	Survey – hematologists participating in GELAMM and additional KOLs	2017–2018	109 (59%) surveys completed	socioeconomic status
Rosso, 2012 [66]	Italy	Italian resident population	WHO database	2008	NR	Age, sex
Salgado, 2019 [67]	US	Patients with MM	NCDB Registry	2004–2014	95,190	Age, sex, race/ethnicity, socioeconomic status, geography
Samy, 2015 [68]	UK	Patients with MM	NCDR, HES	2002–2008	24,361	Age, sex, race/ethnicity, socioeconomic status
Schriber, 2017 [69]	US	Patients with MM, aged 18–75 y, registered for first AHCT	CIBMTR Registry;<br>SEER 18 Registry	2008–2014	24,102 undergoing a first AHCT	Age, sex, race/ethnicity
Siegel, 2017 [70]	US	All cancer patients in the US; subpopulation myeloma	SEER Registry	2009–2013	NR	Sex
Smailyte, 2016 [71]	Lithuania	Patients with first cancer diagnosis	LCR, Statistics Lithuania	2001–2009	926	Sex, Socioeconomic status

Sneyd, 2019 [72]	New Zealand	Patients with first diagnosis of MM	NZCR	1985–2016	7826	Age, sex, race/ethnicity
Sun, 2018 [73]	US	Patients with MM	SEER Registry (9 sites)	1980–2010	2001–2010: 13,088	Age, sex, race/ethnicity, socioeconomic status
Tarín-Arzaga, 2018 [74]	Mexico	Patients with symptomatic NDMM	Medical records, University Hospital, Monterrey	2007–2016	148	Age, socioeconomic status
Tsang, 2019 [75]	Canada	Patients with MM (including 1% with plasma cell leukemia)	CCR, RQC, CVS	1992–2010	32,065	Sex, geography
Uprety, 2017 [76]	US	Elderly patients (over 65 y) diagnosed with MM	SEER 18 Registry	Pre-novel agent era 1991–2002<br>Post-novel agent era 2007–2011	Pre-novel agent era 1991–2002: 13,179<br>Post-novel agent era 2007–2011: 10,139	Age, sex, race/ethnicity
Vanthomme, 2017 [77]	Belgium	Women aged 50–79 y	Belgian census; register data emigration/mortality	2001–2008	NR	Socioeconomic status
Vargas-Serafin, 2021 [78]	Mexico	Patients with NDMM aged ≥18 y	Medical records of single center	2006–2018	245	Age, socioeconomic status
Warren, 2013 [79]	US	Adult patients aged >20 y with newly diagnosed MM	SEER Registry-Patterns of Care	1999, 2003, 2007	Total: 1976<br>1999: 524<br>2003: 710<br>2007: 742	Age, race/ethnicity, socioeconomic status
Wildes, 2018 [80]	US	MM patients<br>Cohort 1: MM at diagnosis<br>Cohort 2: MM post-diagnosis<br>Cohort 3: Non-cancer controls	SEER Registry-MHOS	1998–2011	Cohort 1: MM at Dx, 171<br>Cohort 2: MM post-Dx, 234<br>Cohort 3: Non-cancer controls, 513	Age, sex
Wildes, 2019 [81]	US	Adults aged ≥65 y with newly diagnosed myeloma	Two tertiary care institutions	2012–2015	40	Age, sex, socioeconomic status
Xu, 2020 [82]	China	Patients with NDMM across 9 centers	NR; hospital records assumed	2006–2019	773	Sex, socioeconomic status
Yusuf, 2016 [83]	US	Adult patients (aged >18 y) newly diagnosed with MM and treated with chemotherapeutic agents	Medicare 20% sample	2008–2010	2419	Age, sex, race/ethnicity
Zhou, 2021 [84]	US	Patients with primary MM diagnosis 2001–2011	SEER Registry-Medicare	2001–2013	14,231	Age, sex, race/ethnicity

1L, first-line; ABDS, Australian Burden of Disease Study; AHCT, autologous hematopoietic cell transplantation; ASCT, autologous stem cell transplantation; CCR, Canadian Cancer Registry; CDC, Center for Disease Control and Prevention; CIBMTR, Center for International Blood and Marrow Transplant Research; CoMMpass, Relating Clinical Outcomes in Multiple Myeloma to Personal Assessment of Genetic Profile; CVS, Canadian Vital Statistics; EBMT, European Society for Blood and Marrow Transplantation; Dx, diagnosis; GBD, Global Burden of Disease; GELAMM, Grupo de Estudio Latinoamericano de Mieloma Múltiple; HES, hospital episode statistics; HPC, hematopoietic progenitor cell; INE, Instituto Nacional de Estadística; KOLs, key opinion leaders; LCR, Lithuanian Cancer Registry; MHOS, Medicare Health Outcomes Survey; MM, multiple myeloma; MMRF, Multiple Myeloma Research Foundation; mo, month; MTRH, Moi Teaching and Referral Hospital; N, number; NCCN, National Comprehensive Cancer Network; NCDB, National Cancer Database; NCDR, National Cancer Data Repository; NCI, National Cancer Institute; NCR, National Cancer Registry; NDMM, newly diagnosed multiple myeloma; NHIRD, National Health Institute Research Database; NHS, National Health Survey; NIS, National Inpatient Sample; NR, not reported; NZCR, New Zealand Cancer Registry; PBCR, population-based cancer registry; RMG, Registry of Monoclonal Gammopathies; RQC, le Registre québécois du cancer; RS, relative survival; SEER, Surveillance, Epidemiology, and End Result Program; TPE, therapeutic plasma exchange; US, United States; WCISU, Welsh Cancer Intelligence and Surveillance Unit; WHO, World Health Organization; WUSM, Washington University School of Medicine; y, years.

Supplementary Table 3. Age and disparities in access to treatment in US and non-US studies

Author, year	Outcome	Summary of findings
US studies		
Chhabra, 2020 [18]	Receipt of AHCT	Cumulative incidence of salvage AHCT at 72 mo was age-dependent and highest in those aged 60–64 y age vs the other age groups ($p = 0.006$) (18–59 y: 9.6, 95% CI 4.9–16.1; 60-64 y: 27.0, 95% CI 13.7–42.1; 65–69 y: 10.6, 95% CI 3.8–21.4; ≥70 y: 0)
	Receipt of HPC	Cumulative incidence of HPC utilization for salvage AHCT and HPC boost at 72 mo was age-dependent and higher in those aged 60–64 y vs the other age groups ($p = 0.01$) (18–59 y: 12.7, 95% CI 7.8–20.8; 60–64 y: 30.6, 95% CI 20.1–46.5; 65–69 y: 11.7, 95% CI 5.4–25.2; ≥70 y: 1.1, 95% CI 0.2–7.3)
Costa, 2015 [19]	Uptake of ASCT	AHCT declines with age in RECs for both men and women, with the decline becoming very pronounced >55 y
Fakhri, 2018 [27]	Not in receipt of MM treatment	Older age increased the odds of receiving no treatment (aOR per y 1.07, 95% CI 1.06–1.08)
Fiala, 2015 [28]	Use of SCT	Median age (range) for those who had SCT was 56.9 y (33–74) vs 66.7 y (35–91) for those who did not ($p < 0.001$)
Fiala, 2017 [29]	Use of SCT	Likelihood of receiving SCT decreased with age (aOR per y 0.80, 95% CI 0.78–0.82; $p < 0.0001$)
	Use of bortezomib	Likelihood to use bortezomib decreased with age (aOR per y 0.92, 95% CI 0.92–0.93; $p < 0.0001$)
Fiala, 2020 [30]	Receipt of systemic treatment	Number of patients receiving treatment within 6 mo of diagnosis was significantly lower in those aged ≥80 y (51%) vs those aged 70–79 y (71%; $p < 0.001$)
Fiala, 2020 [31]	Receipt of bortezomib	The likelihood of receiving bortezomib decreased with age (aOR per y 0.94, 95% CI 0.94–0.95; $p < 0.0001$)
	Receipt of lenalidomide	The likelihood of receiving lenalidomide decreased with age (aOR per y 0.95, 95% CI 0.94–0.96; $p < 0.0001$)
Jayakrishnan, 2020 [37]	Delayed time to initial treatment	The odds of treatment delay increased with age (OR 1.01, 95% CI 1.01–1.01; $p < 0.005$)
Kumar, 2020 [44]	Delayed time to initial systemic treatment	Patients received treatment sooner if they were aged ≥80 y (OR 0.83, 95% CI 0.76–0.9; p < 0.001) or 60–79 y (OR 1.1, 95% CI 1.0–1.1; $p = 0.03$) vs 60 y
Pan, 2021 [54]	Collection of stem cells	Greater age at diagnosis was significantly associated with shorter diagnosis-to-collection time (coeff −0.01, Exp [coeff] 0.99, 95% CI 0.98–0.997; $p = 0.007$)
	Between collection of stem cells to transplant	Age at diagnosis did not affect collection-to-transplantation time (coeff 0.00, Exp [coeff] 1.00, 95% CI 0.99–1.02; $p = 0.598$)
Salgado, 2019 [67]	Receipt of EBRT vs no EBRT	Older patients were less likely to receive EBRT (55–64 vs <55 y: OR 0.97, 95% CI 0.91–1.03; $p = 0.3168$; 65–74 vs <55 y: OR 0.97, 95% CI 0.90–1.04; $p = 0.4111$; ≥75 vs <55 y: OR 0.79, 95% CI 0.73–0.85; $p < 0.0001$)
	Receipt of SFRT vs MFRT	Older patients were less likely to receive SFRT than MFRT (55–64 vs <55 y: OR 1.31, 95% CI 0.84–2.06; $p = 0.2355$; 65–74 vs <55 y: OR 1.54, 95% CI 0.95–2.50; $p = 0.0798$; ≥75 vs <55 y: OR 2.11, 95% CI 1.29–3.46; $p = 0.0029$)
Schriber, 2017 [69]	Receipt of AHCT	Fewer Hispanic (39%) and NHB patients (42%) aged >60 y were transplanted vs NHW patients (56%; $p < 0.001$)
Warren, 2013 [79]	Receipt of novel agents	The 50–59 y age group received the highest standardized percentage of novel agents and the ≥70 y age group received the lowest
	Receipt of HSCT	The use of transplantation declined significantly with age
Wildes, 2019 [81]	Receipt of ASCT	Age significantly impacted whether participants underwent ASCT (OR per y 0.77, 95% CI 0.60–0.99; $p = 0.04$)
Zhou, 2021 [84]	Uptake of IV bisphosphonate	Younger age at diagnosis was associated with higher rates of treatment initiation (65–75 y: SHR 1.74, 95% CI 1.59–1.91; 76–85 y: SHR 1.44, 95% CI 1.32–1.58), vs those aged ≥86 y
Non-US studies		
Auner, 2015 [12]	Uptake of AHCT	A smaller proportion of patients aged ≥70 y (70.8%) received AHCT within 1 y of diagnosis vs those aged <40 y (78.5%). Over the study period, the number of AHCTs increased for all age groups, most notably for patients aged ≥65 y (3% of AHCTs in 1991–1995 vs 18.8% in 2006–2010)
Chan, 2020 [15]	OS following funding of bortezomib	For those aged ≤70 y, mOS significantly increased after funding approval for bortezomib for those who did not have ASCT (pre-bortezomib: 49.1 mo, 95% CI 37.1–57.5; post-bortezomib: 62.7 mo, 95% CI 51.7–79.2; $p < 0.01$). No change in mOS over the same time-period for those who received ASCT ($p = 0.81$)
Mian, 2021 [52]	Treatment vs no treatment	Increasing age associated with a lower receipt of treatment (≤65 y: aOR per y 0.98, 95% CI 0.97–1.00; p≤0.05; >65 y: aOR per y 0.91, 95% CI 0.91–0.92, p≤0.05)
	ASCT	Increasing age associated with a lower receipt of transplant (≤65 y: aOR per y 0.93, 95% CI 0.92–0.95; >65 y: aOR per y 0.62, 95% CI 0.59–0.64)
Puyade, 2018 [61]	1L treatment according to guidelines	Those aged ≤65 y were significantly more likely to receive appropriate 1L treatment (95%) vs those aged 66–74 y (93%) and ≥75 y (83%; $p = 0.005$)

Riva, 2019 [64]	Treatment utilization	Younger patients had greater access to novel treatments: 92.5% of those aged ≤70 y received bortezomib or thalidomide vs 50% those aged >70 y.<br>mOS in those treated with novel drugs was not reached vs 22 mo for those who received other treatment options (95% CI 4.92–39.08, $p < 0.001$).<br>1L consolidation with ASCT was performed in 70 patients, all aged <70 y.
Vargas-Serafin, 2021 [78]	Induction therapy	Age was not associated with a decreased likelihood of undergoing induction therapy (≥65 y: OR 1.75, 95% CI 0.82–3.73; $p = 0.143$)

For details on study population(s), data source(s), data period(s), sample size(s) analyzed, and country for non-US studies, see Supplementary Table 2.
1l, first-line; AHCT, autologous hematopoietic cell transplantation; aOR, adjusted odds ratio; ASCT, autologous stem cell transplantation; CI, confidence interval; coeff, coefficient; EBRT, external-beam radiotherapy; Exp, exponential; HPC, hematopoietic progenitor cell; HSCT, hematopoietic stem cell transplant; IV, intravenous; MFRT, multiple-fraction radiotherapy; MM, multiple myeloma; mo, month; mOS, median overall survival; NHB, non-Hispanic Blacks; NHW, non-Hispanic Whites; OR, odds ratio; OS, overall survival; RECs, racial and ethnic categories; SCT, stem cell transplant; SFRT, single-fraction radiotherapy; SHR, sub-distribution hazard ratio; y, year.

Supplementary Table 4. Sex and disparities in access to treatment in US studies

Author, year	Outcome	Summary of findings
Bhatnagar, 2015 [13]	Uptake of ASCT	More Black women than Black men underwent ASCT (*p* < 0.001)
Costa, 2015 [19]	Uptake of ASCT	Higher AHCT utilization in men vs women. 1.3% of potential AHCT procedures are unrealized because of sex disparity, reaching 10.4% among Hispanic patients
Fakhri, 2018 [27]	No MM treatment received	Sex was not associated with treatment receipt status (aOR 1.01, 95% CI 0.87–1.17)
Fiala, 2017 [29]	Use of SCT	Women were less likely to receive SCT than men (aOR 0.81, 95% CI 0.69–0.94; *p* < 0.01)
	Receipt of bortezomib	No difference in use of bortezomib between women and men (aOR 0.98, 95% CI 0.91–1.05; *p* = NS)
Fiala, 2020 [31]	Receipt of bortezomib	Women less likely to receive bortezomib than men (aOR 0.83, 95% CI 0.75–0.93; p = 0.0010)
	Receipt of lenalidomide	No difference in use of lenalidomide between women and men (aOR 0.99, 95% CI 0.88–1.11; *p* = 0.8153)
Jayakrishnan, 2020 [37]	Delayed time to initial anti-myeloma treatment	Women had a higher odds of delayed treatment initiation vs men (OR 1.07, 95% CI 1.01–1.12; *p* < 0.005)
Jayakrishnan, 2021 [38]	Enrollment to systemic therapy	Women had a lower rate of enrollment to systemic therapy vs men (incidence rate ratio 0.94, 95% CI 0.92–0.95; *p* < 0.005)
Kumar, 2020 [44]	Delayed time to initial systemic treatment	Significantly higher likelihood of women being in the group that received delayed treatment (OR 1.15, 95% CI 1.1–1.2; *p* < 0.001)
Pan, 2021 [54]	Collection of stem cells	Sex did not significantly impact access to stem cell collection (coeff 0.01, Exp [coeff] 1.01, 95% CI 0.88–1.16; *p* = 0.845)
	Between collection of stem cells to transplant	Sex did not significantly affect collection-to-transplantation time (coeff 0.01, Exp [coeff] 1.01, 95% CI 0.78-1.32, p = 0.926)
Salgado, 2019 [67]	Receipt of EBRT vs no EBRT	Men were more likely to receive EBRT vs women (OR 1.14, 95% CI 1.01–1.19; *p* < 0.0001)
	Receipt of SFRT vs MFRT	Sex had no impact on receiving SFRT vs MFRT (OR 0.99, 95% CI 0.76–1.27; *p* = 0.082)
Wildes, 2019 [81]	Receipt of ASCT	Sex did not significantly impact receipt of ASCT (OR 0.28, 95% CI 0.073–1.08; *p* = 0.065)
Zhou, 2021 [84]	Uptake of IV bisphosphonate	No significant difference in treatment initiation by sex (SHR 1.05, 95% CI 1.00–1.10)

For details on study population(s), data source(s), data period(s), and sample size(s) analyzed, see Supplementary Table 2.

AHCT, autologous hematopoietic cell transplantation; aOR, adjusted odds ratio; ASCT, autologous stem cell transplantation; CI, confidence interval; coeff, coefficient; EBRT, external-beam radiotherapy; Exp, exponential; IV, intravenous; MFRT, multiple-fraction radiotherapy; MM, multiple myeloma; OR, odds ratio; SCT, stem cell transplant; SFRT, single-fraction radiotherapy; SHR, sub-distribution hazard ratio.

Supplementary Table 5. Race/ethnicity and disparities in access to treatment in US studies

Author, year	Outcome	Summary of findings
Ailawadhi, 2016 [3]	Treatment center/OS	OS improved with access to 2 NCI cancer centers vs none for White but not for non-White patients. OS improved for White and Black patients with access to NCCN cancer centers, but not for Asian or Hispanic patients
Ailawadhi, 2017 [4]	Lenalidomide within first y	Lowest utilization among African American patients (19.4%) and highest among Hispanic patients (23.7%; overall $p < 0.01$). Median d to first dose of lenalidomide after diagnosis was not statistically different by race ($p = 0.14$). Median d of use was lowest among Hispanic (84) and most among Asian patients (112), but was not statistically significant ($p = 0.72$)
	Thalidomide within first y	Significantly higher in use in Hispanic (27.9%) and Asian (28.5%) vs White (18.3%) and African American patients (18.7%; overall $p < 0.01$). Median d to first dose of thalidomide after diagnosis was not statistically significant by race (p = 0.64). Median d of use was lowest in Asian patients (112) vs all other races (140), but was not statistically significant ($p = 0.56$)
	Bortezomib within first y	Significantly different use by race, being lowest among Asian (7.6%) and highest among White patients (12.8%; overall $p < 0.01$). Hispanic patients had the highest median d from diagnosis to first dose (117), while the other groups had a significantly shorter period (median 46–51 d; overall $p = 0.02$).
	SCT withing first y	Significant difference by race, being lowest among Hispanic (1.9%) and highest among White patients (5.8%; overall $p < 0.01$). No significant difference in time to SCT by race ($p = 0.49$)
	Combination therapy within first y (bortezomib only)	Significantly more African American (8.5%) or White patients (8.4%) used bortezomib-only than Hispanic (4.1%) or Asian patients (3.8%; overall $p < 0.01$)
	Combination therapy within first y (IMiD only)	Significantly more Hispanic (42.4%) or Asian patients (40.3%) used lenalidomide or thalidomide vs White (33.3%) or African American patients (31.7%; overall $p < 0.01$)
	Combination therapy within first year (IMiD and bortezomib)	Combined IMiD and bortezomib use was similar across races (White patients: 4.3%; Hispanic patients: 4.1%; African American patients: 3.4%; Asian patients: 3.8%)
Ailawadhi, 2019 [6]	Uptake of novel therapy	Longer median time from diagnosis to novel therapy initiation for African American (5.2 mo) and Hispanic (4.6 mo) vs White patients (2.7 mo; both log-rank $p < 0.05$). Rate of initiating novel therapy was significantly higher for White vs African American (aHR 0.8; $p < 0.05$) and Hispanic patients (aHR: 0.9; $p < 0.05$)
	Uptake of ASCT	Hispanic patients less likely to receive ASCT vs White patients (1-y ASCT rate: 3.2% vs 6.9%; aHR 0.6; $p < 0.05$). No significant difference for African American vs White patients (4.9% vs 6.9%)
Ailawadhi, 2020 [9]	Triplet treatment for 1L induction therapy	Use of treatment was not significantly affected by race (OR 0.98, 95% CI 0.81–1.19; $p = 0.8375$)
	SCT	Similar rates of SCT for African American vs White patients (32% vs 36%; $p = 0.101$)
Ailawadhi, 2021 [10]	Receipt of RT	Black patients less likely to receive RT vs White patients (OR 0.89, 95% CI 0.84–0.93)
Bhatnagar, 2015 [13]	Induction regimen	No difference between Black and White patients ($p = 0.15$)
	Referral for ASCT	Significantly longer delays for Black (1.3 ± 1.5 [SD] y) vs White patients (0.9 ± 1.0 [SD] y; p = 0.003)
	Maintenance therapy	Equal distribution between Black (75%) and White patients (74%)
Costa, 2015 [19]	Uptake of ASCT	Adjusted RUR was significantly higher for NHW (1.17, 95% CI 1.15–1.19) vs NHB (0.69, 95% CI 0.67–0.72; $p < 0.0002$), Hispanic (0.64, 95% CI 0.60–0.69; $p < 0.002$), and Asian patients (0.65, 95% CI 0.58–0.73; $p < 0.0002$). Race and ethnicity disparity prevents 13.8% of AHCT procedures, with a much greater impact seen in NHB, Hispanic, and Asian patients
Derman, 2020 [22]	Induction regimen	Black patients less likely to receive triplet therapies vs White patients (55% vs 73%, $p < 0.001$), including combined PI/IMiD-based (35% vs 46%) or alkylator-based (20% vs 27%) triplet therapy
	Uptake of ASCT	Black patients significantly less likely than White patients to receive 1L ASCT (39% vs 49%, $p = 0.04$) or triplet induction combined with 1L ASCT (33% vs 44%, $p = 0.04$). Of those who received ASCT, there was no racial difference in receiving post-ASCT maintenance therapy
Fakhri, 2018 [27]	No MM treatment received	Those of African American descent were 26% more likely to receive no treatment vs White patients (aOR 1.26, 95% CI 1.03–1.54). Those of Other race were not significantly more likely to receive no treatment vs White patients (aOR 0.95, 95% CI 0.71–1.27)
Fiala, 2015 [28]	SCT utilization	White patients more likely to undergo SCT than Black patients (67% vs 45%, $p < 0.001$)
Fiala, 2017 [29]	Use of SCT	Black patients 37% less likely to undergo SCT vs White patients (aOR 0.63, 95% CI 0.49–0.80; $p < 0.001$)
	Receipt of bortezomib	Black patients 21% less likely to use bortezomib than White patients ($p < 0.0001$)

Fiala, 2020b [31]	Receipt of bortezomib	African American patients 31% less likely to receive bortezomib vs White patients (aOR 0.69, 95% CI 0.59–0.80; $p < 0.0001$). Those of Other race were 21% less likely to receive bortezomib vs White patients (aOR 0.79, 95% CI 0.61–0.81; $p = 0.0453$)
	Receipt of lenalidomide	No difference in lenalidomide use by race (AA vs White patients: aOR 0.89, 95% CI 0.75–1.05; $p = 0.1582$; Other vs White patients: aOR 0.89, 95% CI 0.69–1.13; $p = 0.3267$)
Jayakrishnan, 2020 [37]	Delayed time to initial anti-myeloma treatment	NHB patients had higher odds of delayed time to initial treatment than NHW patients (OR 1.17, 95% CI 1.09–1.25; $p < 0.005$). No significant difference between Hispanic and NHW patients (OR 1.05, 95% CI 0.94–1.17; p = NS)
Jayakrishnan, 2021 [38]	Enrollment to systemic therapy	NHB had lower rates vs NHW patients (incidence rate ratio 0.92, 95% CI 0.90–0.95; $p < 0.005$). No differences for Hispanic vs NHW patients (incidence rate ratio 0.97, 95% CI 0.93–1.0; $p = 0.08$) or Other vs NHW patients
Kumar, 2020 [44]	Delayed time to initial systemic treatment	Significantly higher likelihood of being in the group that received delayed treatment group for NHB vs White patients (OR 1.21, 95% CI 1.14–1.28; $p < 0.001$). No difference for Hispanic or Other vs White patients
Pan, 2021 [54]	Collection of stem cells	Race did not significantly impact access to stem cell collection (White vs African American patients: coeff −0.04, Exp [coeff] 0.96, 95% CI 0.83–1.12; $p = 0.625$)
	Between collection of stem cells to transplant	Time from collection to transplantation for significantly longer for White vs African American patients (coeff 0.28, Exp [coeff] 1.33, 95% CI 1.01–1.74; $p = 0.040$)
Salgado, 2019 [67]	Receipt of EBRT vs no EBRT	White patients more likely to receive EBRT vs Black patients (OR 0.90, 95% CI 0.84–0.95; $p = 0.0002$), but not Hispanic vs White patients (OR 0.94, 95% CI 0.86–1.04; $p = 0.2088$) or Asians/Other vs White patients (OR 0.95, 95% CI 0.83–1.08; $p = 0.4125$)
	Receipt of SFRT vs MFRT	Black patients more likely to receive SFRT vs White patients (OR 1.45, 95% CI 1.03–2.04; $p = 0.0349$), but not Hispanic vs White patients (OR 1.21, 95% CI 0.69–2.10; $p = 0.5103$) or Asians/Other vs White patients (OR 1.44, 95% CI 0.71–2.92; $p = 0.3064$)
Schriber, 2017 [69]	Receipt of AHCT	For those aged >60 y, fewer Hispanic (39%) and NHB patients (42%) were transplanted vs NHW patients (56%; $p < 0.001$). More NHB (50%) and Hispanic women (43%) underwent transplant vs NHW women (41%; $p < 0.001$)
Zhou, 2021 [84]	Uptake of IV bisphosphonate	Non-White patients had significantly delayed or no initiation of treatment vs White patients (Black patients: SHR 0.74, 95% CI 0.70–0.79; API patients: SHR 0.72, 95% CI 0.64–0.82; Hispanics/Latino patients: SHR 0.79, 95% CI 0.72–0.88)

For details on study population(s), data source(s), data period(s), and sample size(s) analyzed, see Supplementary Table 2.

1L, first-line; aHR, adjusted hazard ratio; AHCT, autologous hematopoietic cell transplantation; aOR, adjusted odds ratio; API, Asian/Pacific Islander; ASCT, autologous stem cell transplantation; CI, confidence interval; coeff, coefficient; d, day; EBRT, external-beam radiotherapy; Exp, exponential; IMiD, immunomodulatory drug; IV, intravenous; MFRT, multiple-fraction radiotherapy; MM, multiple myeloma; NCI, National Cancer Institute; NHB, non-Hispanic Blacks; NHW, non-Hispanic Whites; NS, not significant; OR, odds ratio; OS, overall survival; PI, proteasome inhibitor; RT, radiotherapy; RUR, relative utilization ratio; SC, stem cell; SCT, stem cell transplant; SD, standard deviation; SFRT, single-fraction radiotherapy; SHR, sub-distribution hazard ratio; y, year.

Supplementary Table 6. Socioeconomic status and disparities in access to treatment in US and non-US studies

Author, year	Outcome	Summary of findings
US studies		
Chamoun, 2021 [14]	Receipt of HSCT	In those aged <65 y, 33% of those with private insurance received HSCT vs 20% of those on Medicare ($p < 0.0001$). For those aged ≥65 y, 11% of privately insured patients underwent HSCT vs 6% of those on Medicare ($p < 0.0001$)
Fakhri, 2018 [27]	Not in receipt of MM treatment	For every $10,000 increase in median household income, there was no difference in treatment receipt status (aOR 0.98, 95% CI 0.95–1.01). The odds of receiving no treatment were 21% higher in those enrolled in Medicaid in addition to Medicare (aOR 1.21, 95% CI 1.02–1.42)
Fiala, 2015 [28]	Use of SCT	Those with high socioeconomic status (72%) were more likely to undergo SCT than those with middle (59%) or low (52%) status ($p < 0.001$).<br>81% of those with private insurance at diagnosis underwent SCT vs 56% of those with Medicaid, 41% with no insurance, and 31% with Medicare ($p < 0.001$)
Fiala, 2017 [29]	Use of SCT	Likelihood of SCT increased with increasing mean household income (aOR per $10,000 1.08, 95% CI 1.05–1.11; $p < 0.0001$). Those with Medicaid were less likely to receive SCT (aOR 0.49, 95% CI 0.35–0.66; $p < 0.0001$)
	Use of bortezomib	Those in higher-income areas based on mean household income were more likely to use bortezomib (aOR per $10,000 1.05, 95% CI 1.04–1.07; $p < 0.0001$). Those with Medicaid were 23% less likely to use bortezomib (aOR 0.77, 95% CI 0.69–0.86; $p < 0.0001$)
Fiala, 2020 [31]	Receipt of bortezomib	Medicaid enrollment reduced the likelihood of bortezomib use (aOR 0.69, 95% CI 0.60–0.78; $p < 0.0001$)
	Receipt of lenalidomide	Medicaid enrollment reduced the likelihood of lenalidomide use (aOR 0.87, 95% CI 0.75–1.00; $p = 0.0422$)
Jayakrishnan, 2020 [37]	Delayed time to initial treatment	Data reported as not significant
Jayakrishnan, 2021 [38]	Enrollment in systemic therapy	No effect of median income (<$38,000 / $38,000–47,999/ $48,000–62,999/ >$63,000: all *p* = NS). Those in census tracts with more educated residents (7% without high-school education) more likely to be enrolled in systemic treatment than those in census tracts with fewer educated residents (21% without high school education: incidence rate ratio 1.04, 95% CI 1.01–1.07; $p = 0.006$). Uninsured vs private / Medicaid / Medicare/ details unknown: all not significant
Kumar, 2020 [44]	Delayed time to initial systemic treatment	Patient income level not significant under univariate analysis ($p = 0.186$). Patient education level no longer significant under multivariate analysis. Uninsured patients seemed to receive treatment sooner vs those with private insurance (OR 0.81, 95% CI 0.72–0.91; $p < 0.002$); similar results for those with Medicaid vs private insurance (OR, 0.87, 95% CI 0.79–0.95; $p < 0.001$). Medicare vs private insurance, or Other government insurance vs private insurance not significant
Pan, 2021 [54]	Collection of stem cells	Higher socioeconomic status at diagnosis significantly associated with shorter diagnosis-to-collection time (median household income [unit $10,000]: coeff −0.02, Exp [coeff] 0.98, 95% CI 0.96–0.9997; $p = 0.048$)
	Between collection of stem cells to transplant	Higher socioeconomic status at diagnosis trended toward longer collection-to-transplantation times but did not reach significance (median household income [unit $10,000]: coeff 0.04, Exp [coeff] 1.04, 95% CI 0.99–1.08; $p = 0.092$)
Salgado, 2019 [67]	Receipt of EBRT vs no EBRT	Residents in a ZIP code with higher household annual income ($48,000–62,999) more likely to receive EBRT than residents in the lowest household annual income ZIP code (<$38,000: OR 1.11, 95% CI 1.03–1.19; $p = 0.0082$). No difference for residents in the $38,000–47,999 vs <$38,000 ZIP codes (OR 1.05, 95% CI 0.99–1.13; $p = 0.1278$) or the $63,000+ vs <$38,000 ZIP codes (OR 1.05, 95% CI 0.96–1.15; $p = 0.2964$). Residents in a ZIP code with 7% of adults without high school diploma were more likely to receive EBRT than residents in a ZIP code with 21% of adults without high school diploma (OR 1.31, 95% CI 1.19–1.44; $p < 0.0001$). Residents in ZIP code with 13–20.9% of adults without high school diploma also more likely to receive EBRT than the those in the lowest education ZIP code (OR 1.41, 95% CI 1.30–1.52; $p < 0.0001$), but not those in ZIP code with 7–12.9% of adults without high school diploma (OR 1.02, 95% CI 0.94–1.11; $p = 0.6258$). Uninsured less likely to receive EBRT than government insured (OR 1.13, 95% CI 1.01–1.28; $p = 0.0402$), but not privately insured vs government insured (OR 1.03, 95% CI 0.97–1.09; $p = 0.3920$)
	Receipt of SFRT vs MFRT	Annual income, based on household annual income by ZIP code, had no impact on receiving SFRT vs MFRT ($38,000–47,999 vs <$38,000: OR 1.16, 95% CI 0.75–1.80; $p = 0.5007$; $48,000–62,999 vs <$38,000: OR 1.29, 95% CI 0.81–2.05; $p = 0.2856$; $63,000+ vs <$38,000: OR 0.83, 95% CI 0.49–1.41; $p = 0.4905$. Residents living in a ZIP code with <7% of adults without high school diploma were 80% more likely to receive SFRT vs residents in a ZIP code with 21% of adults without high school diploma (OR 1.78, 95% CI 1.06–3.00; $p = 0.0293$). No significant difference between residents of ZIP code with 13–20.9% of adults without high school diploma and residents in ZIP code with 21% of adults without high school diploma (OR 0.81, 95% CI 0.53–1.25; $p = 0.3446$), or residents with 7–12.9% of adults without high school diploma vs residents in ZIP code with 21% of adults without high school diploma (OR 0.90, 95% CI 0.57–1.44; $p = 0.6706$). Insurance status had no impact on receiving SFRT vs MFRT (uninsured vs government insured: OR 1.30, 95% CI 0.64–2.51; $p = 0.4347$; private vs government insured: OR 0.96, 95% CI 0.67–1.37; $p = 0.8186$)

Warren, 2013 [79]	Receipt of novel agents	Among NHW patients, type of insurance was not significantly associated with the receipt of novel agents (Medicare 42.6%; private insurance, 50.2% any Medicaid, 56%). For patients of other races, use of novel agents was significantly lower among those with Medicare only (38.0%) or any Medicaid (36.3%) vs those with private insurance (47.3%)
	Receipt of HSCT	Insurance status not associated with receipt of transplantation for NHW patients, but for patients of other races, transplantation use was significantly lower for any Medicaid or Medicare only, even after adjusting for age ($p \leq 0.001$)
Wildes, 2019 [81]	Receipt of ASCT	Education beyond high school not significantly associated with receipt of ASCT
Non-US studies		
Chan, 2020 [15]	OS following funding of bortezomib	The most deprived groups (deciles 9/10) had an inferior 3-y OS vs other groups (0.57 vs 0.63; $p = 0.026$), and experienced no improvement in survival following the funding of bortezomib
	Uptake of 1L bortezomib, mean cumulative dosage	The most deprived groups (deciles 9/10) had a similar uptake of 1L bortezomib vs other groups ($p = 0.57$), and mean cumulative dosage (65.5 mg vs 71.4 mg; $p = 0.75$)
Intzes, 2020 [36]	Access to ASCT	ASCT offered at a lower percentage to transplant-eligible patients in the low socioeconomic group (25.7%) vs the high socioeconomic group (58.1%; no statistical analysis)
Mian, 2021 [52]	Treatment vs no treatment	For those with low socioeconomic status, both younger and older patients were significantly less likely to receive treatment (≤65 y: aOR 0.77, 95% CI 0.60–0.97; $p \leq 0.05$; >65 y, aOR 0.83, 95% CI 0.72–0.96; $p \leq 0.05$)
	ASCT	No significant association between socioeconomic status and access to ASCT in either younger or older newly patients (≤65 y: aOR 0.81, 95% CI 0.64–1.03; >65 y: aOR 0.88, 95% CI 0.65–1.19)
Riva, 2020 [65]	Access to 1L bortezomib-based triplets	Access for transplant-eligible patients significantly higher for private vs public healthcare (87% vs 52.1%; OR 5.72, 95% CI 2.32–14.7; $p < 0.001$). Access for transplant-ineligible patients significantly higher for private vs public healthcare (86.6% vs 64.4%; OR 4.09, 95% CI 1.57–11.16; $p < 0.01$)
	Access to proteasome inhibitors	Access for all patients (100%) in private centers, but only in 74.7% of public institutions ($p < 0.001$)
	Access to ASCT	For those aged <65 y, access is high (98.2%). ASCT is fully reimbursed in all private and public health institutions. As reported by physicians, time to transplantation is considerably delayed in the public system
Tarin-Arzaga, 2018 [74]	Median number of treatment regimens	Median number of treatment regimens lower for public (3) vs private (5) cohorts. ~70% of those in the private cohort receive further treatment lines with bortezomib (13%), carfilzomib (20%), lenalidomide (32%), or pomalidomide (5%). Those in the public cohort received thalidomide-based regimens exclusively
Vargas-Serafin, 2021 [78]	Induction therapy	Low income not significantly associated with a decreased likelihood of undergoing induction therapy (OR 3.13, 95% CI 0.72–13.69; $p = 0.111$)
Xu, 2020 [82]	Received transplantation	Those with high education levels more likely to receive transplantation vs those with low education levels (59.3% vs 27.9%; $p < 0.001$)
	Received regular treatment	Those with high education levels more likely to undergo regular treatment vs those with low education levels (87.6% vs 60.7%, $p < 0.001$)

For details on study population(s), data source(s), data period(s), sample size(s) analyzed, and country for non-US studies, see Supplementary Table 2.
1L, first-line; aOR, adjusted odds ratio; ASCT, autologous stem cell transplantation; CI, confidence interval; coeff, coefficient; EBRT, external-beam radiotherapy; Exp, exponential; HSCT, hematopoietic stem cell transplantation; MFRT, multiple-fraction radiotherapy; NHW, non-Hispanic Whites; NS, not significant; OR, odds ratio; OS, overall survival; SCT, stem cell transplant; SFRT, single-fraction radiotherapy; y, year; ZIP, Zone Improvement Plan.

Supplementary Table 7. Geography and disparities in access to treatment in US studies

Author, year	Outcome	Summary of findings
Fiala, 2017 [29]	Use of SCT	No difference in receipt of SCT by level of urbanization (urban vs metropolitan: aOR 0.89, 95% CI 0.70–1.12; p = NS; rural vs metropolitan: aOR 1.45, 95% CI 0.88–2.27; p = NS)
	Receipt of bortezomib	No difference in use of bortezomib by level of urbanization (urban vs metropolitan: aOR 0.98, 95% CI 0.89–1.09; p = NS; rural vs metropolitan: aOR 0.78, 95% CI 0.60–1.00; p = NS)
Jayakrishnan, 2020 [37]	Delayed time to initial anti-myeloma treatment	Urban vs metropolitan patients less likely to have delayed treatment initiation (OR 0.91, 95% CI 0.84–0.98; p = 0.015). No significant difference for rural vs metropolitan patients
Jayakrishnan, 2021 [38]	Enrollment to systemic therapy	Urban vs metropolitan residents had a slightly higher incidence of enrollment in systemic therapy (incidence rate ratio 1.05, 95% CI 1.02–1.07; p = 0.001). No significant difference for rural vs metropolitan patients
Kumar, 2020 [44]	Delayed time to initial systemic treatment	Those in the South, Midwest, or West received treatment sooner vs Northeast (all $p < 0.001$)
Salgado, 2019 [67]	Receipt of EBRT vs no EBRT	Those in an urban or rural area more likely to receive EBRT than those in a metropolitan area (urban vs metropolitan: OR 1.26, 95% CI 1.17–1.35; $p < 0.0001$; rural vs metropolitan: OR 1.40, 95% CI 1.12–1.76; p = 0.0043)

For details on study population(s), data source(s), data period(s), and sample size(s) analyzed, see Supplementary Table 2.
aOR, adjusted odds ratio; CI, confidence interval; EBRT, external-beam radiotherapy; NS, not significant; OR, odds ratio; SCT, stem cell transplant.

Supplementary Table 8. Age and disparities in survival in US and non-US studies

Author, year	Outcome	Summary of findings
US studies		
Ailawadhi, 2012 [2]	OS	Increased age at diagnosis was an independent adverse factor associated with decreased OS (18–44 vs ≥75 y: HR 0.320, 95% CI 0.296–0.346; $p < 0.001$; 45–54 vs ≥75 y: HR 0.374, 95% CI 0.357–0.392; $p < 0.001$; 55–64 vs ≥75 y: HR 0.479, 95% CI 0.463–0.497; $p < 0.001$; 65–74 vs ≥75 y: HR 0.656, 95% CI 0.637–0.676; $p < 0.001$). Progressive deterioration in median OS at the age at diagnosis increased across different age cohorts, irrespective of race, sex, or year of diagnosis
	MSS	Increased age at diagnosis was an independent adverse factor associated with decreased MSS (18–44 vs ≥75 y: HR 0.398, 95% CI 0.365–0.435; 45–54 vs ≥75 y: HR 0.458, 95% CI 0.434–0.482; 55–64 vs ≥75 y: HR 0.558, 95% CI 0.535–0.582; 65–74 vs ≥75 y: HR 0.723, 95% CI 0.697–0.749; $p < 0.001$). Progressive deterioration in median MSS at the age at diagnosis increased across different age cohorts, irrespective of race, sex, or year of diagnosis
	5-y RSR	Differences in 5-y RSR were most pronounced in the youngest (18–44 y) and the oldest (>75 y) age cohorts, with Asians doing best and Hispanics the worst, both for OS and MSS
Ailawadhi, 2017 [4]	OS	Survival worsened for every decade increase in patient age (HR 1.71, 95% CI 1.63–1.79; $p < 0.01$)
Ailawadhi, 2018 [5]	OS	Increasing age was associated with inferior median OS (HR per 10-y increase 1.17; $p < 0.001$)
Ailawadhi, 2019 [7]	RSR	In patients aged ≤40 y, 5- and 10-y RSR was highest for NHB and lowest for Hispanics (5-y: Hispanics 56%, NHB 71%, NHW 68%; 10-y: Hispanics 33%, NHB 57%, NHW 46%)<br>In patients aged >40 y, 5- and 10-y RSR were similar across race/ethnicity groups (5-y: Hispanics 45%, NHB 45%, NHW 47%; 10-y: Hispanics 29%, NHB 26%, NHW 28%)
Ailawadhi, 2020 [8]	OS	Age <65 y (HR 0.39, 95% CI 0.34–0.46; $p < 0.0001$) and age 65–75 y (HR 0.55, 95% CI 0.47–0.64; $p < 0.0001$) associated with longer OS vs age ≥75 y
Chamoun, 2021 [14]	OS	Age per y increase was associated with worse survival (all patients: HR 1.044, 95% CI 1.039–1.049; $p = 0.000$; ≥65 y: HR 1.06, 95% CI 1.055–1.068; $p < 0.001$)
Costa, 2016 [20]	OS	Shorter OS with increasing age; older age was associated with increased risk of death (HR per each additional y 1.03, 95% CI 1.02–1.04; $p < 0.001$)
Costa, 2017 [21]	5-y RSR	Improvement in the 5-y RSR was seen among patients aged <65 y (38.2–61.8%; $p < 0.001$), 65–74 y (29.0–48.4%; $p < 0.001$), and ≥75 y (21.1–34.0%; $p < 0.001$)
	10-y RSR	The 10-y RSR compared between 1993–1997 and 2003–2007 (most recent cohort with available 10-y follow-up) showed improvement among patients diagnosed at age <65 y (19.6–35.0%; $p < 0.001$) and 65–74 y (11.7–20.6%; $p < 0.001$), but not among patients aged ≥75 y (7.8–9.3%; p = NS)
	5- and 10-y RSR	An analysis of survival gain by age and race/ethnicity strata indicate notable gains in the 5- and 10-y RSR for patients aged <65 y for all race/ethnicity groups. For patients aged 65–74 y, gains in the 10-y RSR were significant for NHW and Hispanics, but not for NHB. Among patients aged ≥75 y, gains in 5-y RSR were seen for all race/ethnicity groups, whereas improvements in 10-y RSR were not observed for any stratum
Derman, 2020 [22]	PFS	For Black and White patients, age ≥65 y was not associated with PFS (HR 1.2, 95% CI 0.9–1.6; $p = 0.2$)
	OS	For Black and White patients, age ≥65 y was not associated with OS (HR 1.3, 95% CI 0.9–2.0; $p = 0.2$)
Evans, 2021 [26]	OS	Age >75 y was independently associated with worse OS (RR 3.00, 95% CI 2.33–3.88; $p < 0.001$ [utilizing individual socioeconomic risk factors]; RR 3.05, 95% CI 2.36–3.93; $p < 0.001$ [utilizing a cumulative socioeconomic risk score]). mOS for patients with age of diagnosis of <75 vs ≥75 y was 81 vs 26 mo ($p < 0.001$)
Fiala, 2015 [28]	OS	WUSM: age per y was associated with OS (HR 1.02, 95% CI 1.01–1.04; $p = 0.002$)<br>SEER-18: age per y was associated with OS (HR 1.04, 95% CI 1.04–1.04; $p < 0.001$)
Fiala, 2017 [29]	OS	Likelihood to survive decreases with age (aHR per y 1.04, 95% CI 1.03–1.04; $p < 0.0001$)
Fiala, 2020 [30]	OS	Older age per y was associated with shorter survival (≥80 y group: aHR 1.05, 95% CI 1.04–1.07; $p < 0.0001$; 70–79 y group: aHR 1.05, 95% CI 1.03–1.06; $p < 0.0001$)
Hsieh, 2019 [33]	Odds of survival	Younger age at the time of diagnosis was associated with higher odds of long-term survival (60–70 vs <60 y: aOR 0.65, 95% CI 0.60–0.70; $p < 0.001$; >70 vs <60 y: aOR 0.22, 95% CI 0.19–0.24; $p < 0.001$)
Jayakrishnan, 2021 [38]	OS	Risk of death increased with increasing age (HR 1.03, 95% CI 1.027–1.030; $p < 0.0005$)
Kaya, 2012 [42]	OS	The older age groups had worse OS than the <65 y reference group (65–75 y: HR 1.42, 95% CI 1.38–1.46; $p <0.001$; >75 y: HR 2.18, 95% CI 2.12–2.25; $p <0.001$)

Makhani, 2021 [49]	1-y OS	Patients aged ≥80 y had a 4.5-fold increased mortality hazard rate at 1 y (95% CI 3.95−5.23) compared with patients aged 18–49 y. Likewise, ages 50–64 y had an aHR of 1.31 (95% CI 1.13–1.51) and ages 65–79 y had an aHR of 2.18 (95% CI 1.90–2.50) vs ages 18–49 y
	5-y OS	Patients aged <80 y had a 3.9-fold increased mortality hazard rate at 5 y (95% CI 3.58−4.28) vs patients aged 18–49 y. Likewise, ages 50–64 y had an aHR of 1.37 (95% CI 1.25–1.49) and ages 65–79 y had an aHR 2.07 (95% CI 1.90–2.25) vs ages 18–49 y
Patel, 2020 [56]	OS	Risk of death increased with each additional year of age (HR per y 1.03 [1.02–1.03])
Pulte, 2012 [58]	RSR	For patients aged 15–64 y, highest RSR was observed in NHW (52.6%) vs African American s (44.3%) or Hispanics (46.7%). Survival was higher for Hispanics (32.9%) vs NHW (27.1%) among patients aged ≥65 y
Schriber, 2017 [69]	OS	Survival was worse in older age groups (45–60 vs >45 y: HR 1.15, 95% CI 1.02–1.30; *p* = 0.02; 61–75 vs <45 y: HR 1.33, 95% CI 1.18–1.50; *p* < 0.0001)
Sun, 2018 [73]	OS	For patients aged 0–49 y, 50–64 y, 65–79 y, and ≥80 y, survival worsens as age increases (HR 1.042, 95% CI 1.040–1.044; *p* < 0.001)
Uprety, 2017 [76]	RSR	RSR was lower in the ≥80 y group (24.5%, 95% CI 21.5–27.7) vs the 65–79 y group (42.8%, 95% CI 40.8–44.7) and the ≥65 y group (37.9%, 95% CI 36.3–39.6)
Wildes, 2018 [80]	OS	Age was significantly associated with an increased risk of death in patients with newly diagnosed MM (aHR per y 1.05, 95% CI 1.02–1.07, Wald x2 12.18; *p* < 0.001)
Yusuf, 2016 [83]	OS	Older age was significantly associated with increased risk of death (≥65 vs 18–64 y; HR 1.49, 95% CI 1.13–1.96; *p* = 0.005)
Non-US studies		
Chan, 2020 [15]	OS	Age was negatively associated with survival over the whole study period (HR 1.06, 95% CI 1.05 to 1.07)
El Husseiny, 2014 [25]	OS	No statistically significant difference in survival was reported for patients aged ≤50 vs >50 y (*p* > 0.05)
Harwood, 2020 [32]	RSR	A statistically significant decrease in relative survival was reported in older patients
Jones, 2021 [39]	Net survival	For patients diagnosed after 2010, 1-, 5-, and 10-y survival was numerically greater for age <65 vs 65–74 y or 75–90 y
Jurczyszyn, 2016 [40]	OS	Patients aged 21–40 y have a better OS than their counterparts aged 41–60 y, but the survival advantage observed in younger patients was lost in more advanced stages of MM
Kim, 2014 [43]	OS	mOS was longer in patients aged <65 y vs those aged ≥65 y (55 vs 37 mo, *p* < 0.001)
Manyega, 2021 [50]	OS	In univariate analysis, age at diagnosis was not significantly associated with length of survival
Pulte, 2015 [60]	10-y age-standardized RSR	10-y age-standardized RSR decreased with increasing age and was lowest for the oldest age group assessed (65–74 y)
Quaresma, 2015 [62]	Age-adjusted net survival	Net survival was generally lower for the oldest (75–99 y) vs the youngest (15–44 y) patients
Radocha, 2019 [63]	OS	Age above 75 y represents an independent prognostic factor for survival
Riva, 2019 [64]	OS	At a median follow-up of 32 mo, OS was 61.8% (median not reached) in patients aged ≤70 y vs 32 mo (95% CI 22.07–41.93) in patients aged >70 y (*p* < 0.001)
Samy, 2015 [68]	RSR	Relative survival at 1, 3, and 5 y decreased with increasing age
Tarín-Arzaga, 2018 [74]	OS	In multivariate analysis, age was not associated with OS
Vargas-Serafin, 2021 [78]	OS	Age ≥65 y was significantly associated with decreased OS (40 mo vs 49 mo for ages <65 y; *p* = 0.041)

For details on study population(s), data source(s), data period(s), sample size(s) analyzed, and country for non-US studies, see Supplementary Table 2.
aHR, adjusted hazard ratio; aOR, adjusted odds ratio; CI, confidence interval; Dx, Diagnosis; HR, hazard ratio; MM, multiple myeloma; mo, months; mOS, median overall survival; MSS, myeloma-specific survival; NHB, non-Hispanic Black; NHW, non-Hispanic White; NS, not significant; OS, overall survival; PFS, progression-free survival; RR, risk ratio; RSR, relative survival rates; SEER, Surveillance, Epidemiology, and End Result; US, United States; vs, versus; WUSM, Washington University School of Medicine; y, year.

Supplementary Table 9. Age and disparities in mortality in US and non-US studies

Author, year	Outcome	Summary of findings
US studies		
Dhakal, 2020 [24]	In-hospital mortality	Age was not associated with increased in-hospital mortality (≥65 vs 18–64 y: aOR 0.79, 95% CI 0.46–1.35; $p = 0.38$)
Kumar, 2021 [45]	MM-specific early mortality	Advancing age was associated with increased risk of MM-specific early mortality (Q2 [60–68 y] vs Q1 [18–59 y]: OR 1.42, 95% CI 1.33–1.51; $p < 0.001$; Q3 [69–77 y] vs Q1 [18–59 y]: OR 2.3, 95% CI 2.20–2.47; $p < 0.001$; Q4 [≥78 y] vs Q1 [18–59 y]: OR 4.1, 95% CI 3.88–4.33; $p < 0.001$)
	All-cause mortality	Advanced age was associated with increased risk of all-cause mortality (Q2 [60–68 y] vs Q1 [18–59 y]: OR 1.54, 95% CI 1.45–1.62; p < 0.001; Q3 [69–77 y] vs Q1 [18–59 y]: OR 2.5, 95% CI 2.37–2.63; $p < 0.001$; Q4 [≥78 y] vs Q1 [18–59 y]: OR 4.82, 95% CI 4.59–5.07; $p < 0.001$)
Pulte, 2014 [59]	Excess mortality	Older patients had a much higher mortality (15–49 y: 1.00; 50–69 y: EHR 1.49, 95% CI 1.37–1.62; 70 y: EHR 2.64, 95% CI 2.43–2.87)
Non-US studies		
Afshar, 2020 [1]	5-y EMRR	No statistical trend between age at diagnosis and 5-y EMRR ($p = 0.7$)
Chang-Chan, 2021 [16]	Crude rate per 100,000 person-years	Crude mortality rate increased markedly with age (15–49 y: 0.04; 50–69 y: 3.79; ≥70 y: 20.87)
Chen, 2016 [17]	Inpatient mortality	Older vs younger patients had a significantly higher adjusted risk of inpatient mortality (>55 vs ≤55 y, OR: 1.511, 95% CI 1.272–1.796; $p < 0.001$)
Hsu, 2015 [34]	Early mortality	Age ≥70 y not significantly associated with 60-d early mortality in univariate analysis (OR 1.53, 95% CI 0.86–2.72; $p = 0.144$)
Ilic, 2014 [35]	ASMR/100,000 persons	Statistically significant increased mortality among persons aged ≥70 y. Death rates were significantly increased in persons aged 45–69 y
Jones, 2021 [39]	EHR	EHR adjusted for comorbidities was highest in patients aged 75–90 vs 15–64 y at 1, 5 and 10 y after diagnosis
Liu, 2019 [47]	ASMR	After the age of 15 y mortality rates increased steadily with age, with higher rates in those aged >60 y. ASMR had an upward trend with age, peaking in those aged 90–94 y (6.96 [6.20–8.80]/100,000 population)
Mian, 2021 [52]	Early mortality (vs no mortality) among patients receiving novel treatment	Older, but not younger, patients were significantly more likely to experience early mortality vs no early mortality after receipt of novel treatments (≤65 y: aOR 1.00 95% CI 0.98–1.03; >65 y: aOR 1.06 95% CI 1.04–1.07; p≤0.05)
Pastor-Barriuso, 2014 [55]	Mortality rate	Mortality increased with age but showed a gradual deceleration at older ages
Riva, 2019 [64]	Mortality rate	Mortality rate was higher in older patients: 32.9% in those aged <70 y vs 56.6% in those aged >70 y (p = NR)
Rosso, 2012 [66]	ASMR for MM	Death rate from MM was lower for women vs men, and the sex disparity increased with age. Death rate increased with age for both sexes

For details on study population(s), data source(s), data period(s), sample size(s) analyzed, and country for non-US studies, see Supplementary Table 2.
aOR, adjusted odds ratio; d, day; ASMR, age-standardized mortality rate; CI, confidence interval; EHR, excess hazard ratio; EMRR, excess mortality rate ratio; MM, multiple myeloma; NR, not reported; NS, not significant; OR, odds ratio; Q, quartile; y, year.

Supplementary Table 10. Sex and disparities in survival in US and non-US studies

**Author, year**	**Outcome**	**Summary of findings**
US studies		
Ailawadhi, 2012 [2]	OS	Women had significantly better mOS vs men (2.8 vs 2.4 y; HR 0.905, 95% CI 0.882–0.928; $p < 0.001$)
	MSS	Women had significantly better mMSS vs men (3.7 vs 3.6 y; HR 1.037, 95% CI 1.007–1.068; $p = 0.016$)
Ailawadhi, 2017 [4]	OS	An inferior survival was noted for men vs women (HR 1.13, 95% CI 1.06–1.21; $p < 0.01$)
Ailawadhi, 2018 [5]	OS	Women had inferior mOS (HR for men, 0.96; $p = 0.006$)
Ailawadhi, 2019 [6]	RSR	Young women patients had a 10-y RSR of 54% vs 44% in men
Ailawadhi, 2020 [8]	OS	Sex was not associated with longer OS (HR 0.91, 95% CI 0.80–1.03; $p = 0.15$)
Chamoun, 2021 [14]	OS	Sex was not associated with longer OS (all patients: HR 1.06, 95% CI 0.99–1.14; $p = 0.098$; ≥65 y: HR 1.05, 95% CI 0.96–1.15; $p = 0.263$)
Costa, 2016 [20]	OS	Men had shorter OS vs women (HR 1.18, 95% CI 1.10–1.28; $p < 0.001$)
Derman, 2020 [22]	PFS	Male sex was not associated with PFS (HR 1.1, 95% CI 0.9–1.5; $p = 0.4$)
	OS	For the overall cohort, male sex was not associated with OS (HR 1.5, 95% CI 0.97–2.2; $p = 0.07$). In White patients, male sex was associated with inferior OS (HR 1.6, 95% CI 1.0–2.6; $p = 0.047$)
Fiala, 2017 [29]	OS	Women were more likely to survive than men (aHR 0.85, 95% CI 0.81–0.90; $p < 0.0001$)
Fiala, 2020 [30]	OS	Sex had no impact on survival in ≥80 y or 70–79 y age groups (≥80 y: aHR 0.98, 95% CI 0.89–1.08; $p = 0.6330$; 70–79 y: aHR 0.93, 95% CI 0.85–1.03; $p = 0.1577$)
Hsieh, 2019 [33]	Odds of survival	Female sex was associated with higher odds of long-term survival (aOR 1.13, 95% CI 1.05–1.22; $p < 0.001$)
Jayakrishnan, 2021 [38]	OS	Women had a lower risk of death than men (HR 0.9, 95% CI 0.90–0.94; $p < 0.0005$)
Kaya, 2012 [42]	OS	Patient sex did not have a statistically significant impact on survival outcomes
Makhani, 2021 [49]	1-y OS	Women were more likely to survive than men (aHR 0.93, 95% CI 0.88–0.99)
	5-y OS	Women were more likely to survive than men (aHR 0.93, 95% CI 0.89–0.96)
Schriber, 2017 [69]	OS	Women had lower risk of death than men (HR 0.87, 95% CI 0.823–0.92; $p < 0.0001$)
Sun, 2018 [73]	OS	Cox regression analysis of survival between men and women indicated no difference between sexes (HR 0.995, 95% CI 0.951–1.042; $p = 0.839$)
Uprety, 2017 [76]	RSR	There was no difference in RSR by sex (men: 38.3%, 95% CI 36.0–40.6; women: 37.5, 95% CI 35.1–39.9). There was no difference in RSR for any of the race/sex groups (White men: 38.9%, 95% CI 36.2–41.5; White women: 37.0%, 95% CI 34.2–39.8; African American men: 38.2%, 95% CI 32.5–43.9; African American women: 38.7%, 95% CI 33.3–44.0)
Wildes, 2018 [80]	OS	Sex was not significantly associated with risk of death (aHR 0.75, 95% CI 0.51–1.09; Wald x2 2.28, $p = 0.131$)
Yusuf, 2016 [83]	OS	Male sex was significantly associated with increased risk of death (HR 0.86, 95% CI 0.75–0.98; $p = 0.03$)
Non-US studies		
Chan, 2020 [15]	OS	No contribution of sex to survival
El Husseiny, 2014 [25]	OS	No statistically significant difference in survival was reported for men or women ($p > 0.05$)
Harwood, 2020 [32]	RSR	No statistically significant difference was reported for men or women ($p = 0.30$)
	OS	Sex was not significantly associated with 5-y OS (women: 37%, 95% CI 35–39%; men: 36%, 95% CI 35–38%; $p = 0.61$)
Jurczyszyn, 2016 [40]	OS	Sex was not significant (HR 1.18, 95% CI 0.95–1.48; $p = 0.14$)
Kim, 2014 [43]	OS	No significant difference in mOS for men (50 mo) vs women (45 mo; $p = 0.262$)
Manyega, 2021 [50]	OS	Male sex was associated with reduced survival (HR 1.9, 95% CI 1.1–3.3; p = NR)
Pulte, 2015 [60]	10-y age-standardized RSR	10-y age-standardized RSR was slightly higher in women vs men, and most marked in the 45–54 y age group (men: 35.4% [SE: 3.4%]; women: 52.5% [SE: 4.2%])
Puyade, 2018 [61]	OS	No association between sex and survival (HR 1.0, 95% CI 0.7–1.3; $p = 0.86$)
Quaresma, 2015 [62]	Age-adjusted net survival	1-y age-adjusted net survival after diagnosis was higher for men vs women in England (78.0% vs 75.3%) and in Wales (76.2% vs 71.0%)
Radocha, 2019 [63]	OS	Male sex represents an independent survival disadvantage (HR 1.316, 95% CI 1.124–1.541; $p = 0.001$)
Samy, 2015 [68]	RSR	RSR was similar between men and women at 1, 3, and 5 y
Smailyte, 2016 [71]	5-y RSR	5-y RSR (SE) was numerically higher in men vs women for higher education (47.0 [7.3] vs 41.7 [6.2]) but similar for secondary (34.3 [5.7] vs 33.3 [4.8]) and lower than secondary education (26.3 [5.2] vs 26.8 [3.8])
Sneyd, 2019 [72]	Observed survival	Sex was not associated with survival (HR 0.95; $p = 0.120$)

Xu, 2020 [82]	PFS	No significant difference in PFS between men and women
	OS	No significant difference between men and women for OS (HR 1.04, 95% CI 0.83–1.30; $p = 0.74$)

For details on study population(s), data source(s), data period(s), sample size(s) analyzed, and country for non-US studies, see Supplementary Table 2.
aHR, adjusted hazard ratio; aOR, adjusted odds ratio; CI, confidence interval; HR, hazard ratio; mo, months; mOS, median overall survival; MSS, myeloma-specific survival; mMSS, median myeloma-specific survival; NR, not reported; OS, overall survival; PFS, progression-free survival; RSR, relative survival rates; SE, standard error; vs, versus; y, year.

Supplementary Table 11. Sex and disparities in mortality in US and non-US studies

**Author, year**	**Outcome**	**Summary of findings**
US studies		
Costa, 2016 [20]	Early mortality	Greater risk of early mortality for men vs women (HR 1.16, 95% CI 1.02–1.32; *p* = 0.02)
Dhakal, 2020 [24]	In-hospital mortality	Sex was not associated with increased in-hospital mortality (aOR 0.78, 95% CI 0.54–1.15; *p* = 0.21)
Kamath, 2020 [41]	Mortality rate	Mortality rates were varied by sex (White men: 4.0/100,000; White women: 2.4/100,000; Black men: 7.4/ 100,000; Black women: 5.4/100,000)
Kumar, 2021 [45]	MM-specific early mortality	Men were associated with increased risk of MM-specific early mortality (OR 0.95, 95% CI 0.91–0.98; *p* = 0.004)
	All-cause mortality	Men were associated with increased risk of all-cause mortality (OR 0.86, 95% CI 0.86–0.92; *p* < 0.001)
Pulte, 2014 [59]	Excess mortality	No sex difference for excess mortality (men: 1.00; women: EHR 1.05, 95% CI 1.00–1.09)
Siegal, 2017 [70]	Mortality rate ratio	The rate of mortality (per 100,000) was 2.7 for women vs 4.2 for men (ratio 1.6, 95% CI 1.54–1.59)
Non-US studies		
Afshar, 2020 [1]	5-y EMRR	No statistical trend between sex and 5-y EMRR (p-trend = 0.8)
Chang-Chan, 2021 [16]	AMSR per 100,000 person-years	Mortality was markedly higher in men vs women (men: 2.52, 95% CI 2.16–2.89; women 1.85, 95% CI 1.57–2.13)
Chen, 2016 [17]	Inpatient mortality	Significantly higher adjusted risk of inpatient mortality in men vs women (OR: 1.162, 95% CI 1.034–1.305; *p* = 0.012)
Hsu, 2015 [34]	Early mortality	Significantly higher risk of 60-d and 30-d early mortality in men vs women (60-d: aOR 2.71, 95% CI 1.09–6.74; *p* = 0.032; 30-d: aOR 4.49, 95% CI 1.17–17.28; *p* = 0.029)
Liu, 2019 [47]	ASMR	ASMR for men consistently higher than for women (1.5- to 2-fold)
Mian, 2021 [52]	Early mortality (vs no mortality) among patients receiving novel treatment	No significant effect of sex on early mortality vs no early mortality
Pastor-Barriuso, 2014 [55]	Age-adjusted mortality rate	Mortality was higher in men vs women (male:female ratio 1.38, 95% CI 1.31–1.44)
Rosso, 2012 [66]	ASMR for MM	Death rate from MM was lower in women vs men and the sex disparity increased with age. Death rate increased with age for both sexes
Sneyd, 2019 [72]	ASMR	Mortality was higher in Maori men (5.52) vs Maori women (2.58), and non-Maori men (2.83) vs non-Maori women (1.63)
Tsang, 2019 [75]	Total deaths	Numerically more men (52.9%) than women (47.1%) died from MM

For details on study population(s), data source(s), data period(s), sample size(s) analyzed, and country for non-US studies, see Supplementary Table 2.

aOR, adjusted odds ratio; ASMR, age-standardized mortality rate; CI, confidence interval; d, day; EHR, excess hazard ratio; EMRR, excess mortality rate ratio; MM, multiple myeloma; NR, not reported; NS, not significant; OR, odds ratio; Q, quartile; y, year.

Supplementary Table 12. Race/ethnicity and disparities in survival in US and non-US studies

Author, year	Outcome	Summary of findings
US studies		
Ailawadhi, 2012 [2]	OS	Hispanic patients had significantly worse mOS vs White patients (2.4 vs 2.6 y, respectively; HR 1.070, 95% CI 1.021–1.121; $p = 0.006$). Asian patients had a better mOS (2.7 y; HR 0.967, 95% CI 0.908–1.030) and African American patients had worse mOS (2.5 y; HR 1.032, 95% CI 0.995–1.069) vs White patients, but did not reach significance in either group. In those aged ≥75 y, Hispanic patients had significantly worse mOS vs White patients (1.3 vs 1.5 y; HR 1.090, 95% CI 1.002–1.186; $p = 0.002$) while Asian patients had significantly better mOS (1.8 y; HR 0.863, 95% CI 0.778–0.958; $p = 0.002$). mOS in African American patients was similar to White patients (1.6 y; p = NS)
	MSS	Asian patients had the best mMSS among all studied groups (4.1 y) and this was significantly better vs White patients (3.6 y; HR 0.847, 95% CI 0.785–0.914; $p < 0.001$). mMSS was also significantly better in African American patients (3.8 y) vs White patients (HR 0.914, 95% CI 0.875–0.954; $p < 0.001$). Hispanic patients had the worst mMSS (3.5 y) but was not significantly different vs White patients (HR 1.014, 95% CI 0.961–1.070; p = NS). In those aged ≥75 y, Asian patients had the best mMSS (3.3 y) vs White patients (2.4 y; HR 0.743, 95% CI 0.652–0.847; $p < 0.001$), while Hispanic patients had the worst (2.2 y; HR 1.051, 95% CI 0.951–1.162; p = NS vs White patients). African American patients also had significantly better mMSS vs White patients (HR 0.837, 95% CI 0.774–0.905; $p < 0.001$)
	5-y RSR	Differences in 5-y RSR were most pronounced in the youngest (18–44 y) and the oldest (>75 y) cohorts, with Asian patients doing the best and Hispanic patients the worst, both for OS and MSS
Ailawadhi, 2017 [4]	OS	No significant difference in survival was noted by race vs White patients (Hispanic patients: HR 1.08, 95% CI 0.97–1.22; Black patients: HR 1.11, 95% CI 1.01–1.22; Asian patients: HR 1.06, 95% CI 0.91–1.23)
Ailawadhi, 2018 [5]	OS	Compared with White patients, Black patients (HR, 0.86) and Asian patients (HR, 0.88) had better OS ($p < 0.001$)
Ailawadhi, 2019 [6]	RSR	For patients aged ≤40 y, 5- and 10-y RSRs improved significantly over time for NHW and NHB patients (all $p < 0.0001$), but not for Hispanic patients (5-y RSR: Hispanic patients 56%, NHB patients 71%, NHW patients 68%; 10-y RSR: Hispanic patients 33%, NHB patients 57%, NHW patients 46%). For patients aged >40 y, the 5- and 10-y RSR improved significantly for Hispanic, NHB, and NHW patients, and were similar across ethnic groups (5-y RSR: Hispanic patients 45%, NHB patients 45%, NHW patients 47%; 10-y RSR: Hispanic patients 29%, NHB patients 26%, NHW patients 28%)
Ailawadhi, 2019 [7]	OS	mOS was similar among groups (White patients: 2.6 y; African American patients: 2.5 y; Hispanic patients: 2.8 y; log-rank p-values: White vs African American patients, 0.64; White vs Hispanic patients, 0.27). After adjustment, the risk for death was significantly lower for African American vs White patients (aHR, 0.8; $p < 0.05$)
	MSS	mMSS was significantly longer for African American (5.4 y) vs White patients (4.5 y; log-rank $p < 0.05$), and was comparable for Hispanic and White patients (4.9 vs 4.5 y, respectively; log-rank $p = 0.41$)
Ailawadhi, 2020 [8]	OS	Race was not associated with longer OS (Black vs White patients; HR 0.88, 95% CI 0.73–1.07; $p = 0.21$)
Ailawadhi, 2020 [9]	PFS	No significant difference in PFS by race in those undergoing SCT (aHR 0.86, 95% CI 0.65–1.15; $p = 0.3112$) or not undergoing SCT (aHR 0.96, 95% CI 0.82–1.14; $p = 0.6553$)
	OS	For those undergoing SCT, OS was longer in African American vs White patients (aHR 0.56, 95% CI 0.35–0.89; $p = 0.0141$). The adjusted OS in those who did not undergo SCT did not significantly differ by race (HR 0.86, 95% CI 0.70–1.06; $p = 0.1653$)
Bhatnagar, 2015 [13]	PFS with no maintenance therapy	No difference for White vs Black patients who did not receive maintenance therapy (median 1 y for both)
	OS with no maintenance therapy	OS similar for Black patients (4 y, 95% CI 3.1–6.8 y) and White patients (3.7 y, 95% CI 2.3–6.3 y)
Chamoun, 2021 [14]	OS	After adjusting for variables (income, education, area of residence, facility location, comorbidities, previous HSCT, facility type), Black and White patients had similar OS (all patients: HR 1.07, 95% CI 0.98–1.17; $p = 0.124$; ≥65 y: HR 1.05, 95% CI 0.93–1.18; $p = 0.436$)
Costa, 2016 [20]	OS	Hispanic and NHB patients had more adverse sociodemographic factors and worse OS than NHW patients. After adjustment (marital status, insurance status, county-level household income, sex, and age) race/ethnicity was no longer significantly associated with survival (HW vs NHW patients: HR 1.01, 95% CI 0.89–1.14; $p = 0.9$; NHB vs NHW patients: HR 1.01, 95% CI 0.91–1.11; $p = 0.9$; Other vs NHW patients: HR 0.92, 95% CI 0.77–1.10; $p = 0.4$)
Costa, 2017 [21]	5- and 10-y RSR	Notable gains in 5- and 10-y RSR for those aged <65 y for all race/ethnicity groups. For those aged 65–74 y, gains in 10-y RSR were significant for NHW and Hispanic patients, but not for NHB patients. For those aged ≥75 y, gains in 5-y RSR were seen for all race/ethnicity groups, whereas improvements in 10-y RSR were not observed for any stratum. Improvements in survival by sex and race/ethnicity strata indicate gains in 5- and 10-y

		RSR for both sexes and all race/ethnicity groups. Improvements in 5-y RSR were similar in NHW (29.1–50.0%; $p < 0.001$), NHB (32.0–50.1%; $p < 0.001$) and Hispanic patients (29.9–47.3%; $p < 0.001$). Improvements in 10-y RSR were also similar in NHW (13.2–24.3%; $p < 0.001$), NHB (14.6–23.4%; $p < 0.001$), and Hispanic patients (13.0–23.8%; $p = 0.001$)
Derman, 2020 [22]	PFS	Black race was not associated with PFS (HR 1.2, 95% CI 0.8–1.7; $p = 0.3$)
	OS	Black race was not associated with OS (HR 1.4, 95% CI 0.9–2.3; $p = 0.1$). Male sex was associated with inferior OS in White patients (HR 1.6, 95% CI: 1.0–2.6; $p = 0.047$)
Fiala, 2017 [29]	OS	After adjusting for confounders, Black patients had a higher likelihood of survival than White patients (aOR 0.91, 95% CI 0.85–0.97; $p < 0.01$)
Fiala, 2020 [30]	OS	Race had no impact on survival for patients aged ≥80 y (Black vs White patients: aHR 1.01, 95% CI 0.87–1.17; $p = 0.9349$; Other vs White patients: aHR 0.89, 95% CI 0.72–1.09; $p = 0.2479$). For patients aged 70–79 y, race had no impact on survival for Black vs White patients (aHR 0.88, 95% CI 0.77–1.01; $p = 0.0615$). Other patients had better survival than White patients (aHR 0.69, 95% CI 0.56–0.85; $p = 0.0006$)
Hsieh, 2019 [33]	Odds of survival	No significant difference in odds of long-term survival for African American, Native American, or Asian patients vs White patients
Jayakrishnan, 2021 [38]	OS	NHB and Hispanic patients had a lower risk of death than NHW patients (NHB vs NHW patients: HR 0.90, 95% CI 0.87–0.93; $p < 0.0005$; Hispanic vs NHW patients: HR 0.80, 95% CI 0.76–0.85; $p < 0.0005$). No difference between Other and NHW patients (HR 0.97, 95% CI 0.94–1.01; p = NS)
Kaya, 2012 [42]	OS	Asian/Pacific Islander patients had significantly higher OS vs White patients (HR 0.90, 95% CI 0.85–0.96; $p < 0.001$). Black patients had similar OS vs White patients (HR 0.98, 95% CI 0.95–1.01; $p = 0.151$). American Indian/Alaskan Native patients had significantly worse OS vs White patients (HR 1.18, 95% CI 1.01–1.38; $p = 0.040$)
Makhani, 2021 [49]	1-y OS	Black patients had an increased likelihood of survival at 1 y vs White patients (aHR 0.91, 95% CI 0.85−0.98), as did Other patients (aHR 0.82, 95% CI 0.72–0.92). 1-y survival was no different between Hispanic vs non-Hispanic patients (aHR 1.00, 95% CI 0.91–1.09)
	5-y OS	Black patients had an increased likelihood of survival at 5 y vs White patients (aHR 0.92, 95% CI 0.88−0.97), as did Other patients (aHR 0.92, 95% CI 0.85–0.99). 5-y survival was no different between Hispanic vs non-Hispanic patients (aHR 0.99, 95% CI 0.93–1.05)
Patel, 2020 [56]	OS	Black patients had a lower risk of death than White patients (HR 0.85, 95% CI 0.78–0.93)
Pulte, 2012 [58]	RSR	5-y RSR increased significantly for younger NHW patients (+14.9%) and to a smaller degree for older NHW patients (+2.2%). In 1992–1996 in patients aged 15–64 y, 5-y RSR was higher for African American (42.7%) and Hispanic patients (51.6%) vs NHW patients (37.7%). In 2002–2006, the highest RSR was in NHW patients (52.6%) vs African American (44.3%) or Hispanic patients (46.7%). In patients aged ≥65 y, survival was higher for Hispanic vs NHW patients (32.9% vs 27.1%)
Schriber, 2017 [69]	OS	Race had no impact on survival (NHB vs Hispanic patients: HR 0.99, 95% CI 0.89–1.11; $p = 0.2$; NHW vs Hispanic patients: HR 1.07, 95% CI 0.97–1.18; $p = 0.9$)
Sun, 2018 [73]	OS	Univariate analysis of survival for White vs Black/Other patients indicated no differences (HR 1.055, 95% CI 0.996–1.117; $p = 0.069$)
Uprety, 2017 [76]	RSR	RSR was similar for White and African American patients in the 65–79 y group (White patients: 43.0%, 95% CI 40.7–45.2; African American patients: 43.4%, 95% CI 38.9–47.8), and in the ≥80 y group, but lower than the younger group (White patients: 25.1%, 95% CI 21.6–28.8; African American patients: 20.6%, 95% CI 13.3–29.1). There was no difference in RSR between Caucasian and African American patients (38.0%, 95% CI 36.1–40.0 vs 38.6%, 95% CI 34.6–42.5). No differences in RSR for any race/sex groups (White men: 38.9%, 95% CI 36.2–41.5; White women: 37.0%, 95% CI 34.2–39.8; African American men: 38.2%, 95% CI 32.5–43.9; African American women: 38.7%, 95% CI 33.3–44.0)
Yusuf, 2016 [83]	OS	No difference for Black vs White patients (HR 0.90, 95% CI 0.75–1.09; $p = 0.30$) or Other vs White patients (HR 1.20, 95% CI 0.93–1.55; $p = 0.20$)
Non-US studies		
Chan, 2020 [15]	OS	No contribution of ethnicity to survival; mOS was inferior for Maori/Pasifika patients aged ≤70 y but similar for those aged >70 y
Intzes, 2020 [36]	OS	No significant differences in OS by ethnic origin for Greek, Greek Muslim, and Balkan populations
Samy, 2015 [68]	Relative survival	Controlling for age and sex, deprivation, comorbidity, and year of diagnosis, risk of death was significantly lower for Black vs White patients at 1 y and 3 y, and for South Asian vs White patients at 1 y, 3 y, and 5 y
Sneyd, 2019 [72]	Observed myeloma survival	Maori ethnicity was significantly associated with increased hazard for death vs non-Maori ethnicity (HR 1.36; $p < 0.001$ adjusted for age at diagnosis, sex, and year of diagnosis)

For details on study population(s), data source(s), data period(s), and sample size(s) analyzed, and country for non-US studies, see Supplementary Table 2.
aHR, adjusted hazard ratio; aOR, adjusted odds ratio; CI, confidence interval; HR, hazard ratio; HW, Hispanic White; mOS, median overall survival; MSS, myeloma-specific survival; mMSS, median myeloma-specific survival; NHB, non-Hispanic Black; NHW, non-Hispanic White; NR, not reported; NS, not significant; OS, overall survival; PFS, progression-free survival; RSR, relative survival rates; vs, versus; y, year.

Supplementary Table 13. Race/ethnicity and disparities in mortality in US studies

**Author, year**	**Outcome**	**Summary of findings**
Ailawadhi, 2012 [2]	Mortality	For Hispanic vs White patients, 6.5% of mortality could be attributable to ethnicity. For White vs Asian patients, 18% could be attributable to ethnicity
Ailawadhi, 2018 [5]	Death within 30 d of diagnosis	8.8% of White patients had mortality, vs 8.1% of Hispanic, 7.3% of Black, and 7.1% of Asian patients ($p = 0.003$)
Bhatnager, 2015 [13]	TTD	Significantly longer in Black vs White patients (Black patients: 7.7 y, 95% CI 6.5–8.8 y; White patients: 6.1 y, 95% CI 5.2–7.2 y; $p = 0.03$)
DeSantis, 2016 [23]	Rate ratio	In men, mortality rate in NHB patients was 7.8 vs 4.0 in NHW patients (difference: 3.8; ratio: 1.95; $p < 0.05$). In women, mortality rate in NHB patients was 5.4 vs 2.4 in NHW patients (difference: 3.0; ratio: 2.22; $p < 0.05$)
Dhakal, 2020 [24]	In-hospital mortality	Increased mortality in Other/unknown vs White patients (aOR 0.44, 95% CI 0.25–0.78; $p = 0.004$). No effect of race/ethnicity for Black vs White patients (aOR 1.03, 95% CI 0.61–1.74; $p = 0.90$) or Hispanic vs White patients (aOR 0.57,95% CI 0.21–1.53; $p = 0.26$)
Fiala, 2015 [28]	Mortality rate	WUSM: Black patients had lower mortality vs White patients (HR 0.57, 95% CI 0.42–0.76; $p = 0.001$). No significant association between Other and White patients (HR 1.97, 95% CI 0.92–4.24; $p = 0.083$).<br>SEER: Black patients had an increased mortality rate vs White patients (HR 1.09, 95% CI 1.06–1.12; $p < 0.001$). No association between White and Other patients (HR 1.02, 95% CI 0.97–1.07; $p = 0.516$)
Kamath, 2020 [41]	Mortality rate	Mortality rates were lower in White men (4.0/100,000) than Black men (7.4/100,000), and in White women (2.4/100,000) than Black women (5.4/100,000)
Kumar, 2021 [45]	MM-specific early mortality	Increased mortality for NHW vs Other patients (OR 0.79, 95% CI 0.72–0.86; $p < 0.001$), but not vs NHB (OR 0.95, 95% CI 0.9–1.0; $p = 0.06$) or Hispanic patients (OR 1.04, 95% CI 0.97–1.1; $p = 0.60$)
	All-cause mortality	Increased mortality for NHW vs Other patients (OR 0.82, 95% CI 0.76–0.88; $p < 0.001$), but not vs NHB (OR 1.03, 95% CI 0.98–1.08; $p = 0.20$) or Hispanic patients (OR 1.00, 95% CI 0.94–1.07; $p = 0.92$)
Marron, 2018 [51]	Risk of dying	Black patients had 4.13-times the risk of dying vs White patients (95% CI 1.78–9.61; $p = 0.001$)
Pinheiro, 2020 [57]	Rate ratio	African American men had higher mortality rates than White men and other African-descent men. Except for the African group, mortality rate was significantly higher for all other races compared with White patients (African American patients: 1.99, 95% CI 1.51−2.64; Afro-Caribbean patients: 1.82, 95% CI 1.34−2.45; African patients: 1.60, 95% CI 0.85−3.01; Black to White ratio: 1.80, 95% CI 1.36−2.25)<br>For women, except for the African group, mortality was higher for all other races vs White patients (African American patients: 2.41, 95% CI 2.18−2.67; Afro-Caribbean patients: 2.03, 95% CI 1.78−2.32; African patients: 1.39, 95% CI 0.88−2.18, Black to White ratio: 1.94, 95% CI 1.69−2.19)
Pulte, 2014 [59]	Excess mortality	African American and Hispanic patients had a higher excess mortality than NHW patients (African American vs NHW patients: 1.17, 95% CI 1.10–1.23; Hispanic vs NHW patients: 1.14, 95% CI 1.06–1.21; API vs NHW patients: 0.97, 95% CI 0.90–1.07; Other vs NHW patients: 1.03 95% CI 0.85–1.33)

For details on study population(s), data source(s), data period(s), and sample size(s) analyzed, see Supplementary Table 2.

aOR, adjusted odds ratio; API, Asian/Pacific Islander; CI, confidence interval; EHR, excess hazard ratio; HR, hazard ratio; NHB, non-Hispanic Black; NHW, non-Hispanic White; OR, odds ratio; SEER, Surveillance, Epidemiology, and End Result; TTD, time to death; WUSM, Washington University School of Medicine; y, year.

Supplementary Table 14. Socioeconomic status and disparities in survival in US and non-US studies

Author, year	Outcome	Summary of findings
US studies		
Ailawadhi, 2018 [5]	OS	Significant improvement in OS with increasing median income level (adjusted to 2000 Census data). Those with a median income of >$61,600 had significantly better OS vs those with a median income of ≤$34,700 (HR 0.92, 95% CI 0.87–0.98; $p < 0.01$)
Chamoun, 2021 [14]	OS	ZIP-code-based analysis of median income (<$46,000 vs ≥$46,000) showed better OS for higher income in all patients (HR 1.16, 95% CI 1.08–1.25; $p = 0.000$) and in those aged ≥65 y (HR 1.09, 95% CI 1–1.19; $p = 0.064$)<br>In patients aged ≥65 y, median survival was significantly higher in those with private insurance vs Medicare (Private: 41.9 mo, 95% CI 40.1–43.6; Medicare: 30.8 mo, 95% CI 30.3–31.3; $p < 0.0001$). Compared with those with private insurance, the hazard of death was increased by 59% for those with Medicaid, ($p < 0.0001$) and 62% for those with no insurance ($p < 0.0001$). For patients aged ≥65 y, those with no insurance had a 95% higher hazard of death than those with private insurance
Costa, 2016 [20]	OS	Those in the lowest two quartiles of county-level income had a higher risk of mortality vs those in the highest-income quartile (Q1 vs Q4: HR 1.27, 95% CI 1.09–1.49; $p = 0.002$; Q2 vs Q4 HR 1.19, 95% CI: 1.03–1.37; $p = 0.02$; Q3 vs Q4 HR 0.97, 95% CI: 0.85–1.10; $p = 0.6$). County-level education was not associated with OS. Non-insured individuals or those with Medicaid had worse survival than insured individuals (uninsured vs insured: HR 1.43, 95% CI 1.23–1.67; $p < 0.001$; Medicaid vs insured: HR 1.76, 95% CI 1.59–1.94; $p < 0.001$)
Evans, 2021 [26]	OS	Tertiary care center: socioeconomic status risk score ≥1 was independently associated with worse OS (RR 1.36, 95% CI 1.04–1.77; $p = 0.025$)<br>NCDB Registry: low income was associated with worse OS (RR 1.11, 95% CI 1.09–1.14; $p < 0.001$). Residing in non–low-income (Q4) ZIP code vs low-income ZIP codes (Q1, 2, and 3) was associated with higher mOS (57 vs 44 mo; $p < 0.001$). Tertiary care center: low income was not associated with worse OS (RR 1.13, 95% CI 0.899–1.43; $p = 0.322$). Residing in non–low-income vs low-income ZIP codes was associated with higher mOS (80 vs 65 mo; $p = 0.001$)<br>NCDB Registry: low education was associated with worse OS (RR 1.08, 95% CI 1.06–1.11; $p < 0.001$). Residing in ZIP codes of non–low education (Q4) vs low education (Q1, 2, and 3) was associated with higher mOS (56 vs 46 mo; $p < 0.001$). Tertiary care center: low education was not associated with worse OS (RR 1.02, 95% CI 0.88–1.18; $p = 0.765$). Median OS was similar in non–low education vs low education ZIP code areas (70 vs 69 mo; $p = 0.765$)
Fiala, 2015 [28]	OS	WUSM database: low vs high socioeconomic status was associated with worse OS (HR 1.54, 95% CI 1.13–2.09; $p = 0.006$). Differences in OS were significant for middle vs high socioeconomic status (HR 1.25, 95% CI 0.95–1.65; $p = 0.114$). SEER database: the highest socioeconomic status group had significantly better survival vs the low or middle groups (low vs high: HR 1.18, 95% CI 1.15–1.22; $p < 0.001$; middle vs high: HR 1.10, 95% CI 1.07–1.13; $p < 0.001$)<br>WUSM database: no significant association was found between insurance type and OS (Medicare vs private: HR 0.74, 95% CI 0.53–1.03; $p = 0.071$; Medicaid vs private: HR 1.224, 95% CI 0.70–2.14; $p = 0.478$; no insurance vs private: HR 1.59, 95% CI 0.94–2.70; $p = 0.083$)
Fiala, 2017 [29]	OS	Likelihood of survival increased with increasing mean household income (aHR per $10,000 0.97, 95% CI 0.96–0.99; $p < 0.0001$)<br>Those with Medicaid had a greater hazard of death than those without Medicaid (aHR 1.24, 95% CI 1.15–1.33; $p < 0.0001$)
Fiala, 2020 [30]	OS	Medicaid enrollment had no impact on survival in those aged ≥80 y (aHR 1.05, 95% CI 0.94–1.18; $p = 0.4099$). In those aged 70–79 y, Medicaid enrollment was associated with worse survival (aHR 1.25, 95% CI 1.12–1.40; $p < 0.0001$)
Hsieh, 2019 [33]	Odds of survival	Moderately high or high income was associated with higher odds of long-term survival ($35,000–45,999 vs ≥$46,000: aOR 0.98, 95% CI 0.89–1.09; $p = 0.79$; $30,000–34,999 vs ≥$46,000: aOR 0.88, 95% CI 0.77–1.00; $p = 0.06$; <$30,000 vs ≥$46,000: aOR 0.78, 95% CI 0.67–0.91; $p = 0.002$)<br>High educational level was associated with higher odds of long-term survival (middle 2 vs highest: aOR 0.94, 95% CI 0.85–1.05; $p = 0.30$; middle 1 vs highest: aOR 0.88, 95% CI 0.78–0.99; $p = 0.05$; lowest vs highest: aOR 0.81, 95% CI 0.70–0.94; $p = 0.006$)<br>Private insurance was associated with higher odds of long-term survival vs no insurance (aOR 0.57, 95% CI 0.45–0.71; $p < 0.001$)
Jayakrishnan, 2021 [38]	OS	Residents in higher-income census tracts had a lower hazard of death vs those with lower income ($38,000–47,999 vs <$38,000: HR 0.96, 95% CI 0.93–0.99; $p = 0.04$; $48,000–62,999 vs <$38,000: HR 0.93, 95% CI 0.90–0.97; $p < 0.005$; >$63,000 vs <$38,000: HR 0.91, 95% CI 0.87–0.95; $p < 0.005$)<br>Residents of the most educated census tracts had a lower hazard of death than those in the least educated census tracts (13.0–20.9% vs >21.0% [lowest educated]: HR 0.99, 95% CI 0.95–1.02, p = NS; 7.0–12.9% vs >21.0% [lowest educated]: HR 0.98, 95% CI 0.94–1.02; p = NS; <7.0% vs >21.0% [lowest educated]: HR 0.94, 95% CI 0.89–0.99; $p = 0.02$)<br>Those with private insurance or Medicare had a lower risk of death vs the uninsured (private vs uninsured: HR 0.83, 95% CI 0.78–0.89; $p < 0.0005$; Medicare vs uninsured: HR 0.90, 95% CI 0.84–0.96; $p = 0.003$). Those with Medicaid had a higher risk of death vs the uninsured (Medicaid vs

		uninsured: HR 1.09, 95% CI 1.01–1.17; *p* = 0.03). Those with details unknown had a lower hazard of death vs the uninsured (details unknown vs uninsured: HR 0.89, 95% CI 0.81–0.98; *p* = 0.018).
Makhani, 2021 [49]	1-y OS	Those with Medicaid had the highest hazard of 1-y mortality vs private insurance (*p* < 0.001). After adjustment (age, sex, race, ethnicity, marital status, and area of residence) 1-y survival was significantly lower in those with Medicaid vs private insurance (aHR 1.53, 95% CI 1.41−1.67). Those who were uninsured had a 26% increased hazard of mortality vs private insurance (aHR 1.26, 95% CI 1.04−1.53). Insurance not otherwise specified also increased the hazard of mortality (aHR 1.12, 95% CI 1.04–1.20)
	5-y OS	Those with Medicaid had the highest hazard of 5-y mortality vs private insured (*p* < 0.001). After adjustment (age, sex, race, ethnicity, marital status, and area of residence) 5-y survival was significantly lower in those with Medicaid vs private insurance (aHR 1.44, 95% CI 1.36−1.53). Those who were uninsured had a 26% increased hazard of mortality vs private insurance (aHR 1.26, 95% CI 1.11−1.42). Insurance not otherwise specified also increased the hazard of mortality (aHR 1.11, 95% CI 1.06–1.17)
Sun, 2018 [73]	OS	Patients in the low-poverty group had a lower risk of death vs those in the medium/high poverty group (HR 0.879, 95% CI 0.840–0.920; *p* < 0.001)
Non-US studies		
Afshar, 2020 [1]	Net survival	Numerical trend toward decreased net survival in more socioeconomically disadvantaged patients (Q2–Q5) vs less disadvantaged (Q1)
Chan, 2020 [15]	OS	Socioeconomic deprivation was a negative prognostic factor for OS (HR 1.10, 95% CI 1.04–1.16)
Harwood, 2020 [32]	RSR	A statistically significant decrease in RSR was reported in those of disadvantaged socioeconomic status vs affluent, but not for middle class vs affluent
	OS	Patients with disadvantaged socioeconomic status had worse 5-y OS vs those of affluent status (33%, 95% CI 31–36 vs 39%, 95% CI 36–42; *p* = 0.002)
Intzes, 2020 [36]	OS	Low socioeconomic status was associated with worse OS vs high status (HR 2.092, 95% CI 1.36–3.2; *p* = 0.01)
Samy, 2015 [68]	RSR	RSR was numerically greater in more affluent patients at 1, 3, and 5 y
Smailyte, 2016 [71]	5-y RSR	5-y RSR in men and women was highest for those in higher education (men: 20% difference between lower than secondary education vs higher; women: 15% difference between lower than secondary education vs higher). 5-y RSR was numerically higher in men vs women with higher education, but similar for those with secondary and lower than secondary education
Tarin-Arzaga, 2018 [74]	OS	Those who were uninsured had a significantly higher risk of death vs private insurance. Transplant-eligible and transplant-ineligible patient with private healthcare had significantly longer OS vs uninsured
	PFS	Those with private healthcare had significantly longer PFS (*p* < 0.001) vs uninsured. Transplant-eligible and transplant-ineligible patients with private healthcare had significantly longer PFS vs uninsured
Xu, 2020 [82]	PFS	Longer PFS for higher vs lower income (≥42,500 vs <42,500 USD; HR 0.51, 95% CI 0.37–0.70; *p* < 0.001). mPFS was longer in those with high education levels vs low education levels (67.5 vs 30.6 mo; *p* < 0.001). After controlling for clinical and treatment confounders, patients of all ages, as well as either age group, with higher education had lower risk of progression/death vs those with low education<br>Shorter PFS in uninsured vs insured patients (HR 1.54, 95% CI 1.15–2.06; *p* = 0.004)<br>Shorter PFS in unemployed vs employed patients (HR 1.67, 95% CI 1.22–2.30; *p* = 0.002)
	OS	Longer OS for higher vs lower income (≥42,500 vs <42,500 USD; HR 0.36, 95% CI 0.23–0.55; *p* < 0.001)<br>mOS was longer in those with high education levels vs low education levels (122.2 vs 58.83 mo; *p* < 0.001)<br>After controlling for clinical and treatment confounders, patients of all ages, as well as either age group with higher education had lower risk of death vs those with low education<br>Shorter OS in uninsured vs insured patients (HR 2.16, 95% CI 1.43–3.29; *p* < 0.001)<br>Shorter OS in unemployed vs employed patients (HR 2.53, 95% CI 1.55–4.13; *p* < 0.001)

For details on study population(s), data source(s), data period(s), sample size(s) analyzed, and country for non-US studies, see Supplementary Table 2.
aHR, adjusted hazard ratio; aOR, adjusted odds ratio; CI, confidence interval; HR, hazard ratio; mo, month; mOS, median overall survival; mPFS, median progression-free survival; NCDB, National Cancer Database; NS, not significant; OS, overall survival; Q, quartile; RR, risk ratio; SEER, Surveillance, Epidemiology and End Result Program; USD, United States dollars; vs, versus; WUSM, Washington University School of Medicine; y, year; ZIP, Zone Improvement Plan.

Supplementary Table 15. Socioeconomic status and disparities in mortality in US and non-US studies

**Author, year**	**Outcome**	**Summary of findings**
US studies		
Costa, 2016 [20]	Early mortality	Patients in the lowest education quartile (Q1) had a greater risk of early mortality than those in the highest education quartile (Q4; HR 1.34, 95% CI 1.05–1.72; *p* = 0.02). For the middle-income quartiles (Q2 and Q3), early mortality was not different from the highest education quartile (Q2 vs Q4: HR 1.03, 95% CI 0.80–1.32; *p* = 0.84; Q3 vs Q4: HR 0.92, 95% CI 0.72–1.17; *p* = 0.48)<br>Individuals with Medicaid or who were uninsured had higher risk of early mortality than insured individuals (Medicaid vs insured: HR 2.04, 95% CI 1.74–2.41; *p* < 0.001; uninsured vs insured: HR 1.72, 95% CI 1.34–2.19; *p* < 0.001)
Dhakal, 2020 [24]	In-hospital mortality	Insurance was not associated with increased in-hospital mortality (Medicare vs private/HMO: aOR 0.91, 95% CI 0.53–1.57; *p* = 0.73; Medicaid vs private/HMO: aOR 0.43, 95% CI 0.14–1.28; *p* = 0.12; self-pay/other/unknown vs private/HMO: aOR 0.38, 95% CI 0.12–1.29; *p* = 0.12)
Kamath, 2020 [41]	Mortality rate	High mortality rate correlated with average median household income (−0.33; *p* = 0.05), living in poverty (0.44; *p* = 0.01), and food insecurity (0.35; *p* = 0.048), but not with health insurance status (-0.01; *p* = 0.93) or less than high-school education (0.19; *p* = 0.29)
Non-US studies		
Afshar, 2020 [1]	5-y EMRR	Statistically significant increase in EMRR (with 5 y of diagnosis) in the most socioeconomically disadvantaged vs the least disadvantaged patients (*p* = 0.01)
Chen, 2016 [17]	Inpatient mortality	No significant different in mortality between patients living in a household with low-income vs no low-income (OR: 1.613, 95% CI 0.827–3.146; *p* = 0.161)
Lin, 2019 [46]	ASDR	Highest death rates in high-income countries followed by low-income countries (high income: 2.18/100,000, 95% UI 2.61–1.95; low income: 1.28/100,000, 95% UI 1.41–1.13). Highest death rates in most-developed countries, lowest in middle-level developed (most-developed: 2.13/100,000, 95% UI 2.55–1.9; least-developed: 1.03/100,000, 95% UI 1.16–0.95)
Mahmud, 2019 [48]	Fatal cancer burden in 2015	Fatal cancer burden (years of lost life/1000) was highest for those living in the most impoverished areas (0.57) vs the most prosperous (0.51; ratio 1.12; *p* = NR)
Mian, 2021 [52]	Early mortality (vs no mortality) among patients receiving novel treatment	No significant effect of poor socioeconomic status on early mortality in either younger or older patients (≤65 y: aOR 0.99, 95% CI 0.67–1.47; >65 y: aOR 0.93, 95% CI 0.93 0.74–1.18)
Vanthomme, 2017 [77]	ASMR and ASMRR	Mid-level educated women had a mortality rate 18% lower than high-level educated women; there was no difference between low-level and high-level of education (difference -1.6%)<br>All persons (tenants or owners) of lower-comfort housing had a lower mortality rate vs owners of high-comfort housing (difference 34%)

For details on study population(s), data source(s), data period(s), sample size(s) analyzed, and country for non-US studies, see Supplementary Table 2.

aOR, adjusted odds ratio; ASDR, age-standardized death rate; ASMR, age-standardized mortality rate; ASMRR, age-standardized mortality rate ratio; CI, confidence interval; EMRR, excess mortality rate ratio; HMO, Health Maintenance Organization; HR, hazard ratio; NR, not reported; Q, quartile; UI, uncertainty interval; y, year.

Supplementary Table 16. Geography and disparities in survival in US and non-US studies

Author, year	Outcome	Summary of findings
US studies		
Fiala, 2017 [29]	OS	No difference in survival by level of urbanization (urban vs metropolitan: aHR 1.03, 95% CI 0.96–1.10; *p* = NS; rural vs metropolitan: aHR 1.07, 95% CI 0.91–1.27; *p* = NS)
Hsieh, 2019 [33]	Odds of survival	Residency in a rural area vs a metropolitan area was associated with higher odds of long-term survival (aHR 1.48, 95% CI 1.17–1.88; *p* = 0.001). No differences for urban vs metropolitan (aOR 1.04, 95% CI 0.93–1.16; *p* = 0.42)
Jayakrishnan, 2021 [38]	OS	Level of urbanization was not associated with survival (urban vs metropolitan: *p* = NS; rural vs metropolitan: *p* = NS)
Makhani, 2021 [49]	1-y OS	People in urban and rural areas vs metropolitan areas had increased mortality at 1 y (urban vs metropolitan: aHR 1.18, 95% CI 1.07–1.29; rural vs metropolitan: aHR 1.09, 95% CI 0.86–1.37)
	5-y OS	People in urban and rural areas vs metropolitan areas had increased mortality at 5 y (urban vs metropolitan: aHR 1.10, 95% CI 1.03–1.17; rural vs metropolitan: aHR 1.22, 95% CI 1.06–1.42)
Non-US studies		
Chan, 2020 [15]	OS	Differences in survival observed in 4 regions (HR 1.12, 95% CI 1.05–1.19), but regions were not specified
Harwood, 2020 [32]	RSR	A statistically significant increase in RSR was reported in urban vs rural patients
	OS	Urban patients had a superior 5-y OS vs rural patients
Ng, 2020 [53]	OS	No significant difference in OS between patients by remote/rural vs metropolitan residence after adjustment (age, sex, R-ISS, elevated serum LDH, high-risk cytogenetics, ASCT)
Puyade, 2018 [61]	OS	Distance from home to reference center was not significantly associated with survival
Xu, 2020 [82]	PFS	Shorter PFS in rural vs urban patients
	OS	Shorter OS in rural vs urban patients

For details on study population(s), data source(s), data period(s), sample size(s) analyzed, and country for non-US studies, see Supplementary Table 2.

aHR, adjusted hazard ratio; aOR, adjusted odds ratio; ASCT, autologous stem cell transplantation; CI, confidence interval; HR, hazard ratio; LDH, lactate dehydrogenase; NS, not significant; OS, overall survival; PFS, progression-free survival; R-ISS, Revised International Staging System; y, year.

Supplementary Table 17. Geography and disparities in mortality in non-US studies

Author, year	Outcome	Summary of findings
Afshar, 2020 [1]	5-y EMRR	There was a significant trend for increased mortality per quintile increase in socioeconomic disadvantage for urban and rural cases combined, but not significant for urban only
Chen, 2016 [17]	Inpatient mortality	Inpatient mortality risk was significantly higher in urbanized areas vs less urbanized areas
Liu, 2019 [47]	ASMR	Higher mortality rates were clustered in the more developed provinces (highest: Hong Kong Special Administrative Region, Zhejiang, and Shanghai; lowest: Hainan, Fujian, and Shandong)
Mian, 2021 [52]	Early mortality (vs no mortality) among patients receiving novel treatment	No significant association between urban vs rural location and mortality in either younger or older patients
Tsang, 2019 [75]	Mortality rate	Mortality rates in Nova Scotia (42.59, 95% CI 39.61–45.74) and Saskatchewan (39.34, 95% CI 36.59–42.25) were significantly higher, and rates in Alberta (27.85, 95% CI 26.52–29.23) and Northern Territories (13.03, 95% CI 8.43–19.23) were significantly lower, vs the national average (35.77, 95% CI 35.29–36.26)

For details on study population(s), data source(s), data period(s), sample size(s) analyzed, and country see Supplementary Table 2.

ASMR, age-standardized mortality rate; CI, confidence interval; EMRR, excess mortality rate ratio; y, year.

References

1. Afshar, N, English, DR, Blakely, T, Thursfield, V, Farrugia, H, Giles, GG, et al. Differences in cancer survival by area-level socio-economic disadvantage: A population-based study using cancer registry data. PLoS One. 2020; 15:e0228551.
2. Ailawadhi, S, Aldoss, IT, Yang, D, Razavi, P, Cozen, W, Sher, T, et al. Outcome disparities in multiple myeloma: a SEER-based comparative analysis of ethnic subgroups. Br J Haematol. 2012; 158:91–98.
3. Ailawadhi, S, Advani, P, Yang, D, Ghosh, R, Swaika, A, Roy, V, et al. Impact of access to NCI- and NCCN-designated cancer centers on outcomes for multiple myeloma patients: a SEER registry analysis. Cancer. 2016; 122:618–625.
4. Ailawadhi, S, Frank, RD, Advani, P, Swaika, A, Temkit, Mh, Menghani, R, et al. Racial disparity in utilization of therapeutic modalities among multiple myeloma patients: a SEER-medicare analysis. Cancer Med. 2017; 6:2876–2885.
5. Ailawadhi, S, Frank, RD, Sharma, M, Menghani, R, Temkit, M, Paulus, S, et al. Trends in multiple myeloma presentation, management, cost of care, and outcomes in the Medicare population: a comprehensive look at racial disparities. Cancer. 2018; 124:1710–1721.
6. Ailawadhi, S, Parikh, K, Abouzaid, S, Zhou, Z, Tang, W, Clancy, Z, et al. Racial disparities in treatment patterns and outcomes among patients with multiple myeloma: a SEER-Medicare analysis. Blood Adv. 2019; 3:2986–2994.
7. Ailawadhi, S, Azzouqa, AG, Hodge, D, Cochuyt, J, Jani, P, Ahmed, S, et al. Survival trends in young patients with multiple myeloma: a focus on racial-ethnic minorities. Clin Lymphoma Myeloma Leuk. 2019; 19:619–623.

8. Ailawadhi, S, Jagannath, S, Narang, M, Rifkin, RM, Terebelo, HR, Toomey, K, et al. Connect MM Registry as a national reference for United States multiple myeloma patients. Cancer Med. 2020; 9:35–42.
9. Ailawadhi, S, Jagannath, S, Lee, HC, Narang, M, Rifkin, RM, Terebelo, HR, et al. Association between race and treatment patterns and survival outcomes in multiple myeloma: a Connect MM Registry analysis. Cancer. 2020; 126:4332–4340.
10. Ailawadhi, S, Frank, R, Ailawadhi, M, Kanji, Z, Jani, P, Fiala, M, et al. Utilization of radiation therapy in multiple myeloma: trends and changes in practice. Ann Hematol. 2021; 100:735–741.
11. Auner, HW, Pavlu, J, Szydlo, R, Giles, C, Kanfer, E, Macdonald, D, et al. Autologous haematopoietic stem cell transplantation in multiple myeloma patients from ethnic minority groups in an equal access healthcare system. Br J Haematol. 2012; 157:125–127.
12. Auner, HW, Szydlo, R, Hoek, J, Goldschmidt, H, Stoppa, AM, Morgan, GJ, et al. Trends in autologous hematopoietic cell transplantation for multiple myeloma in Europe: increased use and improved outcomes in elderly patients in recent years. Bone Marrow Transplant. 2015; 50:209–215.
13. Bhatnagar, V, Wu, Y, Goloubeva, OG, Ruehle, KT, Milliron, TE, Harris, CG, et al. Disparities in black and white patients with multiple myeloma referred for autologous hematopoietic transplantation: a single center study. Cancer. 2015; 121:1064–1070.
14. Chamoun, K, Firoozmand, A, Caimi, P, Fu, P, Cao, S, Otegbeye, F, et al. Socioeconomic factors and survival of multiple myeloma patients. Cancers (Basel). 2021; 13:590.

15. Chan, HSH, Milne, RJ. Impact of age, sex, ethnicity, socio-economic deprivation and novel pharmaceuticals on the overall survival of patients with multiple myeloma in New Zealand. Br J Haematol. 2020; 188:692–700.

16. Chang-Chan, DY, Rios-Tamayo, R, Rodriguez Barranco, M, Redondo-Sanchez, D, Gonzalez, Y, Marcos-Gragera, R, et al. Trends of incidence, mortality and survival of multiple myeloma in Spain. A twenty-three-year population-based study. Clin Transl Oncol. 2021; 23:1429–1439.

17. Chen, JH, Chung, CH, Wang, YC, Hsu, SN, Huang, WY, Chien, WC. Prevalence and mortality-related factors of multiple myeloma in Taiwan. PLoS One. 2016; 11:e0167227.

18. Chhabra, S, Thapa, B, Szabo, A, Konings, S, D'Souza, A, Dhakal, B, et al. Utilization and cost implications of hematopoietic progenitor cells stored for a future salvage autologous transplantation or stem cell boost in myeloma patients. Biol Blood Marrow Transplant. 2020; 26:2011–2017.

19. Costa, LJ, Huang, JX, Hari, PN. Disparities in utilization of autologous hematopoietic cell transplantation for treatment of multiple myeloma. Biol Blood Marrow Transplant. 2015; 21:701–706.

20. Costa, LJ, Brill, IK, Brown, EE. Impact of marital status, insurance status, income, and race/ethnicity on the survival of younger patients diagnosed with multiple myeloma in the United States. Cancer. 2016; 122:3183–3190.

21. Costa, LJ, Brill, IK, Omel, J, Godby, K, Kumar, SK, Brown, EE. Recent trends in multiple myeloma incidence and survival by age, race, and ethnicity in the United States. Blood Adv. 2017; 1:282–287.

22. Derman, BA, Jasielec, J, Langerman, SS, Zhang, W, Jakubowiak, AJ, Chiu, BC-H. Racial differences in treatment and outcomes in multiple myeloma: a multiple myeloma research foundation analysis. Blood Cancer J. 2020; 10:80.

23. DeSantis, CE, Siegel, RL, Sauer, AG, Miller, KD, Fedewa, SA, Alcaraz, KI, et al. Cancer statistics for African Americans, 2016: progress and opportunities in reducing racial disparities. CA Cancer J Clin. 2016; 66:290–308.

24. Dhakal, B, Miller, S, Rein, L, Pathak, LK, Gloria, L, Szabo, A, et al. Trends in the use of therapeutic plasma exchange in multiple myeloma. J Clin Apher. 2020; 35:307–315.

25. El Husseiny, NM, Kasem, N, El Azeeim, HA, Mattar, MW. Multiple myeloma: a descriptive study of 217 Egyptian patients. Ann Hematol. 2014; 93:141–145.

26. Evans, LA, Go, R, Warsame, R, Nandakumar, B, Buadi, FK, Dispenzieri, A, et al. The impact of socioeconomic risk factors on the survival outcomes of patients with newly diagnosed multiple myeloma: a cross-analysis of a population-based registry and a tertiary care center. Clin Lymphoma Myeloma Leuk. 2021; 21:451–460.

27. Fakhri, B, Fiala, MA, Tuchman, SA, Wildes, TM. Undertreatment of older patients with newly diagnosed multiple myeloma in the era of novel therapies. Clin Lymphoma Myeloma Leuk. 2018; 18:219–224.

28. Fiala, MA, Finney, JD, Liu, J, Stockerl-Goldstein, KE, Tomasson, MH, Vij, R, et al. Socioeconomic status is independently associated with overall survival in patients with multiple myeloma. Leuk Lymphoma. 2015; 56:2643–2649.

29. Fiala, MA, Wildes, TM. Racial disparities in treatment use for multiple myeloma. Cancer. 2017; 123:1590–1596.

30. Fiala, MA, Foley, NC, Zweegman, S, Vij, R, Wildes, TM. The characteristics, treatment patterns, and outcomes of older adults aged 80 and over with multiple myeloma. J Geriatr Oncol. 2020; 11:1274–1278.

31. Fiala, MA, Gettinger, T, Wallace, CL, Vij, R, Wildes, TM. Cost differential associated with hospice use among older patients with multiple myeloma. J Geriatr Oncol. 2020; 11:88–92.

32. Harwood, M, Dunn, N, Moore, J, Mollee, P, Hapgood, G. Trends in myeloma relative survival in Queensland by treatment era, age, place of residence, and socioeconomic status. Leuk Lymphoma. 2020; 61:721–727.

33. Hsieh, RW, Go, RS, Abeykoon, JP, Kapoor, P, Kumar, SK, Gertz, MA, et al. Characteristics of long-term survivors with multiple myeloma: a National Cancer Data Base analysis. Cancer. 2019; 125:3574–3581.

34. Hsu, P, Lin, TW, Gau, JP, Yu, YB, Hsiao, LT, Tzeng, CH, et al. Risk of early mortality in patients with newly diagnosed multiple myeloma. Medicine (Baltimore). 2015; 94:e2305.

35. Ilic, M, Ilic, I. Malignant lymphatic and hematopoietic neoplasms mortality in Serbia, 1991-2010: a joinpoint regression analysis. PLoS One. 2014; 9:e109379.

36. Intzes, S, Symeonidou, M, Zagoridis, K, Bezirgiannidou, Z, Pentidou, A, Vrachiolias, G, et al. Socioeconomic status is an independent prognostic factor for overall survival in patients with multiple myeloma: real-world data from a cohort of 223 patients. Clin Lymphoma Myeloma Leuk. 2020; 20:704–711.

37. Jayakrishnan, T, Bakalov, V, Callander, NS, Sadashiv, S, Wagner, R, Ailawadhi, S. Impact of the Affordable Care Act on timeliness to treatment for patients with multiple myeloma. Anticancer Res. 2020; 40:5727–5734.

38. Jayakrishnan, TT, Bakalov, V, Chahine, Z, Lister, J, Wegner, RE, Sadashiv, S. Disparities in the enrollment to systemic therapy and survival for patients with multiple myeloma. Hematol Oncol Stem Cell Ther. 2021; 14:218–230.

39. Jones, A, Bowcock, S, Rachet, B. Survival trends in elderly myeloma patients. Eur J Haematol. 2021; 106:126–131.

40. Jurczyszyn, A, Nahi, H, Avivi, I, Gozzetti, A, Niesvizky, R, Yadlapati, S, et al. Characteristics and outcomes of patients with multiple myeloma aged 21-40 years versus 41-60 years: a multi-institutional case-control study. Br J Haematol. 2016; 175:884–891.

41. Kamath, GR, Renteria, AS, Jagannath, S, Gallagher, EJ, Parekh, S, Bickell, NA. Where you live can impact your cancer risk: a look at multiple myeloma in New York City. Ann Epidemiol. 2020; 48:43–50. e44.

42. Kaya, H, Peressini, B, Jawed, I, Martincic, D, Elaimy, AL, Lamoreaux, WT, et al. Impact of age, race and decade of treatment on overall survival in a critical population analysis of 40,000 multiple myeloma patients. Int J Hematol. 2012; 95:64–70.

43. Kim, K, Lee, JH, Kim, JS, Min, CK, Yoon, SS, Shimizu, K, et al. Clinical profiles of multiple myeloma in Asia-An Asian Myeloma Network study. Am J Hematol. 2014; 89:751–756.

44. Kumar, V, Alhaj-Moustafa, M, Bojanini, L, Sher, T, Roy, V, Manochakian, R, et al. Timeliness of initial therapy in multiple myeloma: trends and factors affecting patient care. JCO Oncol Pract. 2020; 16:e341–e349.

45. Kumar, V, Ailawadhi, M, Dutta, N, Abdulazeez, M, Aggarwal, CS, Quintero, G, et al. Trends in early mortality from multiple myeloma: a population-based analysis. Clin Lymphoma Myeloma Leuk. 2021; 21:e449–e455.

46. Lin, L, Yan, L, Liu, Y, Yuan, F, Li, H, Ni, J. Incidence and death in 29 cancer groups in 2017 and trend analysis from 1990 to 2017 from the Global Burden of Disease Study. J Hematol Oncol. 2019; 12:96.

47. Liu, J, Liu, W, Mi, L, Zeng, X, Cai, C, Ma, J, et al. Incidence and mortality of multiple myeloma in China, 2006-2016: an analysis of the Global Burden of Disease Study 2016. J Hematol Oncol. 2019; 12:136.

48. Mahumud, RA, Alam, K, Dunn, J, Gow, J. Emerging cancer incidence, mortality, hospitalisation and associated burden among Australian cancer patients, 1982 - 2014: an incidence-based approach in terms of trends, determinants and inequality. BMJ Open. 2019; 9:e031874.

49. Makhani, SS, Shively, D, Castro, G, Rodriguez de la Vega, P, Barengo, NC. Association of insurance disparities and survival in adults with multiple myeloma: a non-concurrent cohort study. Leuk Res. 2021; 104:106542.

50. Manyega, KM, Lotodo, TC, Oduor, MA, Namaemba, DF, Omondi, AA, Oyolo, YL, et al. Retrospective analysis of presentation, treatment, and outcomes of multiple myeloma at a large public referral hospital in Eldoret, Kenya. JCO Glob Oncol. 2021; 7:391–399.

51. Marron, MM, Ives, DG, Boudreau, RM, Harris, TB, Newman, AB. Racial differences in cause-specific mortality between community-dwelling older black and white adults. J Am Geriatr Soc. 2018; 66:1980–1986.

52. Mian, HS, Seow, H, Wildes, TM, Kouroukis, CT, Pond, GR, Sivapathasundaram, B, et al. Disparities in treatment patterns and outcomes among younger and older adults with newly diagnosed multiple myeloma: a population-based study. J Geriatr Oncol. 2021; 12:508–514.

53. Ng, TF, Burrow, S, Leahy, M, Augustson, B, Trentino, K, De Kraa, R, et al. Patients with multiple myeloma over a large catchment of 2.5 million square kilometres: a Western Australia retrospective survival review. Intern Med J. 2020; 50:869–872.

54. Pan, D, Coltoff, A, Ozbek, U, Lin, JY, Afshar, S, Galitzeck, Z, et al. Evaluating race and time to transplantation in multiple myeloma: the Mount Sinai hospital experience. Clin Lymphoma Myeloma Leuk. 2021; 21:439–443.

55. Pastor-Barriuso, R, Lopez-Abente, G. Changes in period and cohort effects on haematological cancer mortality in Spain, 1952-2006. BMC Cancer. 2014; 14:250.

56. Patel, BG, Luo, S, Wildes, TM, Sanfilippo, KM. Frailty in older adults with multiple myeloma: a study of US veterans. JCO Clin Cancer Inform. 2020; 4:117–127.

57. Pinheiro, PS, Medina, H, Callahan, KE, Kwon, D, Ragin, C, Sherman, R, et al. Cancer mortality among US blacks: variability between African Americans, Afro-Caribbeans, and Africans. Cancer Epidemiol. 2020; 66:101709.

58. Pulte, D, Redaniel, M, Brenner, H, Jeffreys, M. Changes in survival by ethnicity of patients with cancer between 1992–1996 and 2002–2006: is the discrepancy decreasing? Ann Oncol. 2012; 23:2428–2434.

59. Pulte, D, Redaniel, MT, Brenner, H, Jansen, L, Jeffreys, M. Recent improvement in survival of patients with multiple myeloma: variation by ethnicity. Leuk Lymphoma. 2014; 55:1083–1089.

60. Pulte, D, Jansen, L, Castro, FA, Emrich, K, Katalinic, A, Holleczek, B, et al. Trends in survival of multiple myeloma patients in Germany and the United States in the first decade of the 21st century. Br J Haematol. 2015; 171:189–196.

61. Puyade, M, Defossez, G, Guilhot, F, Leleu, X, Ingrand, P. Age-related health care disparities in multiple myeloma. Hematol Oncol. 2018; 36:224–231.

62. Quaresma, M, Coleman, MP, Rachet, B. 40-year trends in an index of survival for all cancers combined and survival adjusted for age and sex for each cancer in England and Wales, 1971-2011: a population-based study. The Lancet. 2015; 385:1206–1218.

63. Radocha, J, Hájek, R, Brožová, L, Pour, L, Špička, I, Minařík, J, et al. Simplified novel prognostic score for real-life older adults with multiple myeloma-registry-based analysis. Ann Hematol. 2019; 98:951–962.

64. Riva, E, Bove, V, Villano, F, Mori, M, Cordoba, C, Noria, A, et al. From guidelines to real world: results from the National Multiple Myeloma Registry in Uruguay on 222 newly diagnosed multiple myeloma patients from 2012 to 2015. Curr Med Res Opin. 2019; 35:1197–1203.

65. Riva, E, Schütz, N, Peña, C, Ruiz-Argüelles, G, Hopkins, CR, Bove, V, et al. Significant differences in access to tests and treatments for multiple myeloma between public and private systems in Latin America. Results of a Latin American survey. GELAMM (Grupo de Estudio Latino Americano de Mieloma Múltiple). Ann Hematol. 2020; 99:1025–1030.

66. Rosso, T, Malvezzi, M, Bertuccio, P, Negri, E, La Vecchia, C, Decarli, A. Cancer mortality in Italy, 2008, and predictions for 2012. Tumori. 2012; 98:559–567.

67. Salgado, LR, Chang, S, Ru, M, Moshier, E, Ghiassi-Nejad, Z, Lazarev, S, et al. Utilization patterns of single fraction radiation therapy for multiple myeloma. Clin Lymphoma Myeloma Leuk. 2019; 19:e238–e246.

68. Samy, EF, Ross, J, Bolton, E, Morris, EJ, Oliver, SE. Variation in incidence and survival by ethnicity for patients with myeloma in England (2002-2008). Leuk Lymphoma. 2015; 56:2660–2667.

69. Schriber, JR, Hari, PN, Ahn, KW, Fei, M, Costa, LJ, Kharfan-Dabaja, MA, et al. Hispanics have the lowest stem cell transplant utilization rate for autologous

hematopoietic cell transplantation for multiple myeloma in the United States: a CIBMTR report. Cancer. 2017; 123:3141–3149.

70. Siegal, R, Miller, K, Jemal, A. Cancer statistics, 2017. CA Cancer J Clin. 2017; 67:7–30.

71. Smailyte, G, Jasilionis, D, Vincerzevskiene, I, Shkolnikov, VM. Education, survival, and avoidable deaths in Lithuanian cancer patients, 2001-2009. Acta Oncol. 2016; 55:859–864.

72. Sneyd, MJ, Cox, B, Morison, IM. Trends in myeloma incidence, mortality and survival in New Zealand (1985-2016). Cancer Epidemiol. 2019; 60:55–59.

73. Sun, T, Wang, S, Sun, H, Wen, J, An, G, Li, J. Improved survival in multiple myeloma, with a diminishing racial gap and a widening socioeconomic status gap over three decades. Leuk Lymphoma. 2018; 59:49–58.

74. Tarín-Arzaga, L, Arredondo-Campos, D, Martínez-Pacheco, V, Martínez-González, O, Ramírez-López, A, Gómez-De León, A, et al. Impact of the affordability of novel agents in patients with multiple myeloma: real-world data of current clinical practice in Mexico. Cancer. 2018; 124:1946–1953.

75. Tsang, M, Le, M, Ghazawi, FM, Cyr, J, Alakel, A, Rahme, E, et al. Multiple myeloma epidemiology and patient geographic distribution in Canada: a population study. Cancer. 2019; 125:2435–2444.

76. Uprety, D, Adhikari, J, Arjyal, L, Naglak, MC, Seidman, M. Racial differences in the survival of elderly patients with multiple myeloma in pre-and post-novel agent era. J Geriatr Oncol. 2017; 8:125–127.

77. Vanthomme, K, Vandenheede, H, Hagedoorn, P, Gadeyne, S. Evolution of socioeconomic inequalities in site-specific cancer mortality among Belgian women

between 1991 and 2008 using a fundamental cause approach. Cancer Causes Control. 2017; 28:829–840.

78. Vargas-Serafin, C, Acosta-Medina, AA, Ordonez-Gonzalez, I, Martinez-Banos, D, Bourlon, C. Impact of socioeconomic characteristics and comorbidities on therapy initiation and outcomes of newly diagnosed multiple myeloma: real-world data from a resource-constrained setting. Clin Lymphoma Myeloma Leuk. 2021; 21:182–187.

79. Warren, JL, Harlan, LC, Stevens, J, Little, RF, Abel, GA. Multiple myeloma treatment transformed: a population-based study of changes in initial management approaches in the United States. J Clin Oncol. 2013; 31:1984–1989.

80. Wildes, TM, Fiala, MA. Falls in older adults with multiple myeloma. Eur J Haematol. 2018; 100:273–278.

81. Wildes, TM, Tuchman, SA, Klepin, HD, Mikhael, J, Trinkaus, K, Stockerl-Goldstein, K, et al. Geriatric assessment in older adults with multiple myeloma. J Am Geriatr Soc. 2019; 67:987–991.

82. Xu, L, Wang, X, Pan, X, Wang, X, Wang, Q, Wu, B, et al. Education level as a predictor of survival in patients with multiple myeloma. BMC Cancer. 2020; 20:737.

83. Yusuf, AA, Natwick, T, Werther, W, Felici, D, Mahue, M, Bridges, KR, et al. A retrospective analysis to examine factors associated with mortality in Medicare beneficiaries newly diagnosed with multiple myeloma. Curr Med Res Opin. 2016; 32:1989–1996.

84. Zhou, J, Sweiss, K, Nutescu, EA, Han, J, Patel, PR, Ko, NY, et al. Racial disparities in intravenous bisphosphonate use among older patients with multiple myeloma enrolled in medicare. JCO Oncol Pract. 2021; 17:e294–e312.
